# Production of ammonia makes Venusian clouds habitable and explains observed cloud-level chemical anomalies

**DOI:** 10.1073/pnas.2110889118

**Published:** 2021-12-20

**Authors:** William Bains, Janusz J. Petkowski, Paul B. Rimmer, Sara Seager

**Affiliations:** ^a^Department of Earth, Atmospheric, and Planetary Sciences, Massachusetts Institute of Technology, Cambridge, MA 02139;; ^b^School of Physics & Astronomy, Cardiff University, Cardiff CF24 3AA, United Kingdom;; ^c^Department of Earth Sciences, University of Cambridge, Cambridge CB2 3EQ, United Kingdom;; ^d^Cavendish Laboratory, University of Cambridge, Cambridge CB3 0HE, United Kingdom;; ^e^Medical Research Council, Laboratory of Molecular Biology, Cambridge CB2 0QH, United Kingdom;; ^f^Department of Physics, Massachusetts Institute of Technology, Cambridge, MA 02139;; ^g^Department of Aeronautics and Astronautics, Massachusetts Institute of Technology, Cambridge, MA 02139

**Keywords:** Venus, clouds, atmospheric chemistry, astrobiology, habitability

## Abstract

The atmosphere of Venus remains mysterious, with many outstanding chemical connundra. These include the unexpected presence of ∼10 ppm O_2_ in the cloud layers, an unknown composition of large particles in the lower cloud layers, and hard to explain measured vertical abundance profiles of SO_2_ and H_2_O. We propose a hypothesis for the chemistry in the clouds that largely addresses all of the above anomalies. We include ammonia (NH_3_), a key component that has been tentatively detected both by the Venera 8 and Pioneer Venus probes. NH_3_ dissolves in some of the sulfuric acid cloud droplets, effectively neutralizing the acid and trapping dissolved SO_2_ as ammonium sulfite salts. This trapping of SO_2_ in the clouds, together with the release of SO_2_ below the clouds as the droplets settle out to higher temperatures, explains the vertical SO_2_ abundance anomaly. A consequence of the presence of NH_3_ is that some Venus cloud droplets must be semisolid ammonium salt slurries, with a pH of ∼1, which matches Earth acidophile environments, rather than concentrated sulfuric acid. The source of NH_3_ is unknown but could involve biological production; if so, then the most energy-efficient NH_3_-producing reaction also creates O_2,_ explaining the detection of O_2_ in the cloud layers. Our model therefore predicts that the clouds are more habitable than previously thought, and may be inhabited. Unlike prior atmospheric models, ours does not require forced chemical constraints to match the data. Our hypothesis, guided by existing observations, can be tested by new Venus in situ measurements.

## Significance

This research provides a transformative hypothesis for the chemistry of the atmospheric cloud layers of Venus while reconciling decades-long atmosphere anomalies. Our model predicts that the clouds are not entirely made of sulfuric acid, but are partially composed of ammonium salt slurries, which may be the result of biological production of ammonia in cloud droplets. As a result, the clouds are no more acidic than some extreme terrestrial environments that harbor life. Life could be making its own environment on Venus. The model’s predictions for the abundance of gases in Venus’ atmosphere match observation better than any previous model, and are readily testable.

Venus is often called Earth’s sister planet because of its similar mass and size to Earth. Yet, owing, in part, to the greenhouse effect from its massive CO_2_ atmosphere, Venus’s surface temperature is higher than 700 K—too hot for life of any kind. The Venusian surface is therefore a complete contrast to Earth’s temperate surface and rich surface biosphere. Nonetheless, scientists have been speculating on Venus as a habitable world for over half a century (1–7). Such speculations are based on the Earth-like temperature and pressure at the altitudes of 48 km to 60 km above the surface (8, 9).

Venus is perpetually shrouded in an ∼20-km-deep layer of clouds, including the temperate atmosphere layers at 48 km to 60 km. The prevailing consensus is that the clouds of Venus are made from droplets of concentrated sulfuric acid. This conclusion is inferred from the presence of small amounts of sulfuric acid vapor in the atmosphere (10, 11) and the refractive index of cloud droplets (12, 13). While the clouds are often described as “temperate” or “clement,” such a statement is misleading when it comes to habitability. If the cloud particles are actually made of concentrated sulfuric acid, then it is difficult to imagine how life chemically similar to life on Earth could survive (7, 14). Specifically, the aggressive chemical properties of sulfuric acid and the extremely low atmospheric water content (14, 15) are orders of magnitude more acidic and 50 to 100 times drier than any inhabited extreme environment on Earth.

## Overview of Venusian Atmosphere Anomalies

Despite over 50 y of remote and local observation, Venus’s atmosphere has a number of lingering anomalies with either poor model fits or no explanations (16, 17).

One such long-standing mysterious feature of the atmosphere, which is not well explained by current atmospheric chemistry models, is the abundance profile of water vapor and SO_2_ in and above the cloud layers (17–19).

Observations show that H_2_O persists throughout the atmosphere, while the SO_2_ is observed in parts per million abundances below the clouds and parts per billion abundance above the clouds. Yet, expectations are very different. The primary source of SO_2_ and H_2_O in the atmosphere of Venus is volcanism. As the gases are released from volcanoes, they are uniformly mixed vertically throughout the atmosphere. At very high altitudes in the atmosphere, around 70 km, SO_2_ and H_2_O are efficiently destroyed by ultraviolet (UV) radiation. However, the observed SO_2_ and H_2_O abundance profiles deviate from the uniform distribution, notably, such that SO_2_ shows significant depletion in the cloud layers and H_2_O is present above the cloud layers.

Previous consensus models explained the SO_2_ profile by suggesting that SO_2_ is photochemically oxidized to SO_3_, which then reacts with water to form sulfuric acid in the clouds:

$$\begin{array}{l} {\text{CO}}_{2}+h\nu \to \text{CO}+\text{O}\\ {\text{SO}}_{2}+\text{O}+\text{M}\to {\text{SO}}_{3}+\text{M}\\ {\text{SO}}_{3}+{\text{2H}}_{2}\text{O}\to {\text{H}}_{2}{\text{SO}}_{4}+{\text{H}}_{2}\text{O}\\ {\text{SO}}_{2}+{\text{H}}_{2}\text{O}+{\text{CO}}_{2}\to {\text{H}}_{2}{\text{SO}}_{4}+\text{CO.} \end{array}$$

However, as there is 5× more SO_2_ than H_2_O, this chemistry should strip all the water out of the cloud layer, and additionally react with and prevent water from reaching and accumulating above the clouds as well, while only reducing SO_2_ by 20%, not the 99.9% observed (20). Previous models provide a numerical fix to match the observations, arbitrarily removing SO_2_ or artificially keeping the water abundance constant (21, 22).

Another mystery is the presence of O_2_ in the clouds (23, 24), as there is no known process for O_2_ formation in the cloud layers (discussed further below). Finally, the SO_2_, O_2_, and H_2_O anomalies, together with other trace atmospheric gas abundances, form a chemical disequilibrium in the clouds of Venus (25–27).

A more tentative but intriguing anomaly is that of the detection of NH_3_ in and below the cloud layers. NH_3_ was tentatively detected both by the Venera 8 chemical probe (28) and in reanalyzed Pioneer Venus (Pioneer 13) data (27). The reanalysis of Pioneer Venus data showed additional N species (NO_x_), suggesting further chemical disequilibrium in the cloud layers. The cloud particles themselves also contain many unknowns. The largest particles, predominant in the lower cloud decks [called Mode 3 particles (29)], may have a substantial solid component, implying that they cannot be exclusively made of liquid concentrated sulfuric acid (30).

Some additional anomalies that are not directly relevant to this work, such as the “unknown UV absorber” (31) and the possible presence of methane (32) or phosphine (33, 34), have all been suggested as signs of life in the clouds.

## How the Rimmer et al. Model Resolves the SO_2_ and H_2_O Abundance Conundrum

Recently, Rimmer et al. (20) proposed a mechanism to explain the depletion of SO_2_ in the atmospheric cloud layers, as well as the vertical abundance profile of H_2_O in and above the clouds. If a base is present inside the cloud sulfuric acid droplets, SO_2_ will dissolve in the liquid droplets (by reaction with OH^−^) to form sulfite. The base (B), therefore traps the SO_2_ inside the cloud droplet as sulfite (HSO_3_^−^),

$${\text{SO}}_{2}+{\text{H}}_{2}\text{O}+\text{B}\to {\text{SO}}_{2}+{\text{BH}}^{+}+{\text{OH}}^{-}\to {\text{BH}}^{+}+{\text{HSO}}_{3}{}^{-}.$$

In summary, the equilibrium of the reaction

$${\text{SO}}_{2}+{\text{H}}_{2}\text{O}↔{\text{H}}_{2}{\text{SO}}_{3}$$

is pulled to the right of the above equation, and S(IV) species are trapped as sulfite salts through reaction with the base. Thus, SO_2_ is depleted in the cloud layer, compared to the model with no bases. Eventually, the cloud droplets rain down to lower atmosphere layers, and the salts dissociate due to higher temperatures, releasing SO_2_.

Water is consumed in the sulfite-forming reaction, but is recycled into the lower atmosphere on breakdown of the sulfites, which provides a mechanism to explain the water vapor abundance profile through the clouds. Some water is removed from the cloud layer, but, because it is replenished by recycling from below the clouds, the water removal is not absolute, and so some water remains at the cloud top and in the atmosphere above the clouds. Thus the Rimmer et al. (20) model predicts that both SO_2_ and H_2_O will be present above the clouds but at substantially lower abundance than they are below the clouds, in agreement with observation.

The formation of the sulfite salt within a droplet effectively neutralizes the acid in the droplet, with the very important outcome that some of the cloud droplets are much less acidic than previously thought, with a pH between −1 and 1 (20), instead of an acidity of approximately −11 (on the Hammett acidity scale). If correct, the revised pH range of some droplets has a significance for the habitability of the clouds of Venus that cannot be overstated. Such a pH range is habitable by terrestrial extremophiles (35), as compared to the acidity of concentrated sulfuric acid in which all terrestrial life, and most terrestrial biochemicals, would be destroyed (14).

We argue that the identity of any droplet-neutralizing base is unknown. Rimmer et al. (20) adopted NaOH as a model base for their calculations, but noted that iron oxides are a more physically realistic possibility. In principle, minerals that can absorb SO_2_ could be delivered to the clouds from Venusian volcanic eruption, from wind lofting of dust, or from meteoritic infall. However, it has not been demonstrated that such mechanisms could deliver the very high amount of ∼20 tonnes per second flux of mineral salts (specifically iron oxides) required (20).

We are motivated to extend ref. 20’s analysis with the hypothesis that the neutralizing base that is capturing SO_2_ is locally generated in the clouds. We postulate that NH_3_ is the neutralizing agent for the Venusian cloud droplets, trapping SO_2_ and thus explaining the drop in SO_2_ abundance across the clouds. We are additionally motivated by the tentative in situ observations of NH_3_ in the Venus cloud layers, from both Venera 8 chemical assay (28) and Pioneer Venus probe mass spectrometry (27). If present, NH_3_ observations cannot yet be readily explained through any known abiotic planetary processes (36). We therefore also explore the possibility that the NH_3_ is biologically produced.

## Results

### Ammonia as a Neutralizing Agent in the Venusian Cloud Droplets.

We propose NH_3_ as the only plausible neutralizing base that can be generated in situ in the clouds from gas-phase components (see *SI Appendix*, section 1 for further details on potential neutralizing agents in the cloud layers). The presence of NH_3_, as with any neutralizing base, leads to chemistry that results in the SO_2_ depletion in the clouds and the observed H_2_O abundance profile, and is consistent with a subset of Mode 3 particles being nonspherical (i.e., not liquid) and not composed of pure concentrated sulfuric acid. The presence of NH_3_ may also solve the otherwise unexplained presence of O_2_ in the clouds, especially if the source of NH_3_ is biological.

To support our hypothesis that NH_3_ could explain the presence of O_2_ within the clouds, we first explore the limited number of possible chemical reactions that could lead to the formation of NH_3_ in the Venusian atmosphere cloud layer conditions (Table 1).

**Table 1.** Free energy per mole for NH_3_-generating reactions under Venus cloud conditions

	Reaction	Free energy of reaction (kJ/mol)	Free energy required per mole of surplus NH_3_ (kJ/mol)	Water consumed per surplus NH_3_
1	4N_2(aq)_ + 11H_2_O_(l)_ → 2NH_4_^+^OH^−^_(aq)_ + 3NH_4_^+^NO_3_^−^_(aq)_	1,730 to 2,024	865 to 1,012	6.5
2	N_2(aq)_ + 8H_2_O_(l)_ → 2NH_4_^+^OH^−^_(aq)_ + 3H_2_O_2(aq)_	1,203 to 1,471	602 to 736	4
**3**	**2N_2(aq)_ + 10H_2_O_(l)_ → 4NH_4_^+^OH^−^_(aq)_ + 3O_2(aq)_**	**1,000 to 1,306**	**262 to 343**	**2.5**
4	4N_2(aq)_ + 17H_2_O_(l)_ + 3HCl_(aq)_ → 5NH_4_^+^OH^−^_(aq)_ + 3NH_4_^+^ClO_4_^−^_(aq)_	1,364 to 1,634	273 to 323	3.4
5	N_2(aq)_ + 6H_2_O_(l)_ + 3SO_2(aq)_ → (NH_4_^+^)_2_SO_4_^2-^_(aq)_ + 2H_2_SO_4(aq)_	1,193 to 1,313	N/A	N/A

Free energies of NH_3_-producing reactions are calculated from refs. 83–85. Ranges are minimum to maximum over a range of pH = −3 to pH = +4 and temperature from 2 °C to 115 °C. Concentrations of SO_2_ and H_2_O are as described in ref. 34. O_2_ fractional abundance is assumed to be 10^−6^. Table columns are as follows. First column: reaction number. Second column: possible chemical reaction that produces NH_3_. Third column: free energy of reaction assuming that NH_3_ is accumulated to 2 molar concentration. For the fourth and fifth columns, values were calculated in terms of “surplus NH_3_,” which is the amount of NH_3_ synthesized as NH_4_OH. Fourth column: free energy per mole of “surplus NH_3_” produced. Fifth column: number of water molecules consumed per “surplus” NH_3_. Reaction 3 (bold type), which produces molecular oxygen as an oxidized byproduct, is the most efficient, in both its use of energy and its use to water. We note that reaction 4 could produce hypochlorite, chlorite, or chlorate as an oxidized product, but, as perchlorate is relatively stable and is the weakest oxidizing agent, we have shown this reaction for illustration only. Reaction 5 generates more acid than it consumes, and so cannot be a source of the base which neutralizes H_2_SO_3_. We also note that reaction 1 and reaction 4 (reactions making nitrate and perchlorate, respectively) clouds also alternatively explain the presence of O_2_. Nitrate and perchlorate would “rain out” and decompose to N_2_ and O_2_ or HCl, Cl_2_, and O_2_, respectively, below the clouds. In situ measurements of NO_x_ and ClO_4_ abundance in the clouds could rule out these reactions as a potential source of indirect formation of O_2_.

The most abundant source of nitrogen atoms in the atmosphere of Venus is N_2_ gas, so, to make NH_3_, N_2_ must be reduced to NH_3_. The reduction of N_2_ to form NH_3_ requires a source of hydrogen atoms, and a source of electrons (reducing equivalents). Hydrogen atoms are rare in the atmosphere of Venus. The most abundant gas-phase source of hydrogen atoms in the atmosphere of Venus is H_2_O, followed by HCl. In order to generate reducing equivalents, some species must be oxidized. Species available to be oxidized include CO, OCS, SO_2_, N_2_, H_2_O, and HCl. Phosphorus, if present, will be overwhelmingly present as H_3_PO_4_ (34); neither H_3_PO_4_ or CO_2_ can be further oxidized.

The most energy- and water-efficient NH_3_-producing reaction (reaction 3 in Table 1) also produces molecular oxygen. We choose reaction 3, 2N_2(aq)_ + 10H_2_O_(l)_ → 4NH_4_^+^OH^−^_(aq)_ + 3O_2(aq)_, as the basis for our model for two reasons, Firstly, parsimony leads us to prefer a reaction that uses the smallest amount of rare materials (H_2_O and energy). Secondly, reaction 3 is the only NH_3_-forming reaction that directly produces O_2_ in the clouds (Table 1), whose detection is one of the anomalies we wish to explain (discussed below); the other reactions produce different oxidized species which would not be observed but which would also produce O_2_ on breakdown, and at the cost of greater energy consumption.

A key question is what NH_3_ production rate (by reaction 3) is needed for maintaining the low SO_2_ abundance, as compared to expected equilibrium values in the atmospheric cloud layers. We base the SO_2_ production rate on the rate at which SO_2_ would be replenished into the clouds by mixing from below, and hence the rate at which it must be removed from the clouds. The flux is ∼10^11^ tonnes per year NH_3_, which is on the order of photosynthetic production of O_2_ on Earth (see *Materials and Methods*). This flux is calculated assuming that NH_3_ is only produced to sequester SO_2_, and that only NH_3_ sequesters SO_2_. If other species contribute to removing SO_2_, whether hydroxide salts, iron oxides, or other species, the NH_3_ production will be accordingly lower. Any byproduct of SO_2_ sequestration must have a flux of ∼10^11^ tonnes per year at the bottom of the clouds, based on the SO_2_ depletion within the clouds. A flux of 10^11^ tonnes per year is consistent, to within an order of magnitude, with the mass loss at the bottom of the clouds from rainout of Mode 3 particles from our calculations (*SI Appendix*, section 2).

All of the NH_3_-producing reactions in the Venusian atmosphere conditions are highly endergonic (Table 1), and so must be coupled to an energy source if the reactions are to produce net, “surplus” NH_3_. There are several energy sources that could, in principle, drive the production of NH_3_. Lightning falls short by many orders of magnitude of the necessary rate of production of NH_3_ (*SI Appendix*, section 7.1 and Table S3), and is very unlikely to produce both NH_3_ and O_2_ simultaneously. Similarly, UV photochemistry is unlikely to produce NH_3_ in more than trace amounts (*SI Appendix*, section 7.2), although we note that the photochemistry of nitrogen species in concentrated sulfuric acid has not been explored. Volcanic sources of NH_3_ on Earth are closely associated with organic deposits, including coal, and also are quantitatively insufficient, even based on terrestrial rates of volcanic NH_3_ production, which are likely to be much higher than any plausible NH_3_ production on Venus (*SI Appendix*, section 7.3 and Fig. S4).

The ability to couple chemical energy to drive endergonic reactions is a universal characteristic of life, and, specifically, the use of energy to drive the reduction of N_2_ to NH_3_ in an oxidizing environment is widely found in terrestrial organisms (37, 38). We should therefore consider the possibility that living organisms in the clouds of Venus are making NH_3_. All of the NH_3_-producing reactions presented in Table 1 consume water, which is a rare resource in the clouds of Venus. The energy expended and water molecules consumed in the process of making NH_3_ must be balanced by an equally powerful benefit to the organism for this apparently wasteful chemistry. Neutralizing the acid to make the droplets habitable is a clear benefit.

**Fig. 1.**
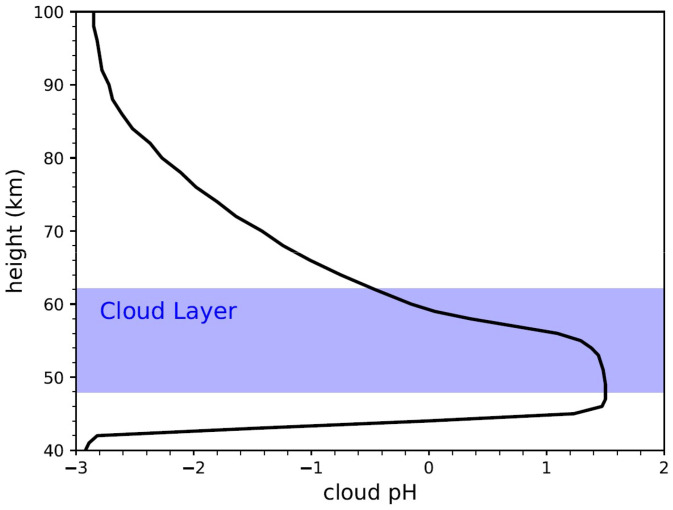
Predicted pH profile of cloud particles. The blue shaded region shows the altitude where clouds are present, from 48 km to 62 km. Note that the plot extends above and below the cloud tops because there are plausibly cloud particle populations that extend down to the altitude where sulfuric acid is sublimated, and up into the mesosphere where sulfuric acid aerosol evaporation may explain the anomalous SO_2_ inversion at 80 km to 100 km. Our model provides no constraints on the composition of the mesospheric particles, which may well be composed of pure sulfuric acid.

We discuss the other, possibly insuperable barriers to the concept of life in the Venusian clouds below. Here we only note that the presence of life could explain the observed presence of NH_3_ and O_2_, and later show that it could explain the observed vertical abundances of H_2_O and SO_2_ within and above the atmospheric cloud layers, and the semisolid nature of Mode 3 particles. An additional consequence of the NH_3_ cloud droplet chemistry is that the pH of cloud particles with dissolved NH_3_ must have a pH between −1 and 1, as first shown by Rimmer et al. (20) for NaOH (Fig. 1).

### The Flux of NH_3_ Is within the Plausible Biomass Production.

The flux of NH_3_ needed to achieve the neutralization effect is not prohibitive for a realistic biomass within the cloud droplets. We calculate the biomass required by this model as follows. The production of 10^11^ tonnes per year is equivalent to 3·10^9^ g NH_3_ per second. Several species of cyanobacteria fix nitrogen at an average rate of ∼4·10^−7^ g per g wet weight biomass per second (39–41). If life is present in the clouds of Venus, it will not be terrestrial life; however, if we take these terrestrial organisms as precedent, 10^11^ tonnes per year would be produced by ∼8·10^15^ g wet weight of organism. While this mass might appear significantly high, it is ∼1/2,000 (0.05%) the biomass of the Earth (42). This mass translates to ∼1.5% of the mass of cloud particles in the lower 5 km of the cloud deck (25).

**Fig. 2.**
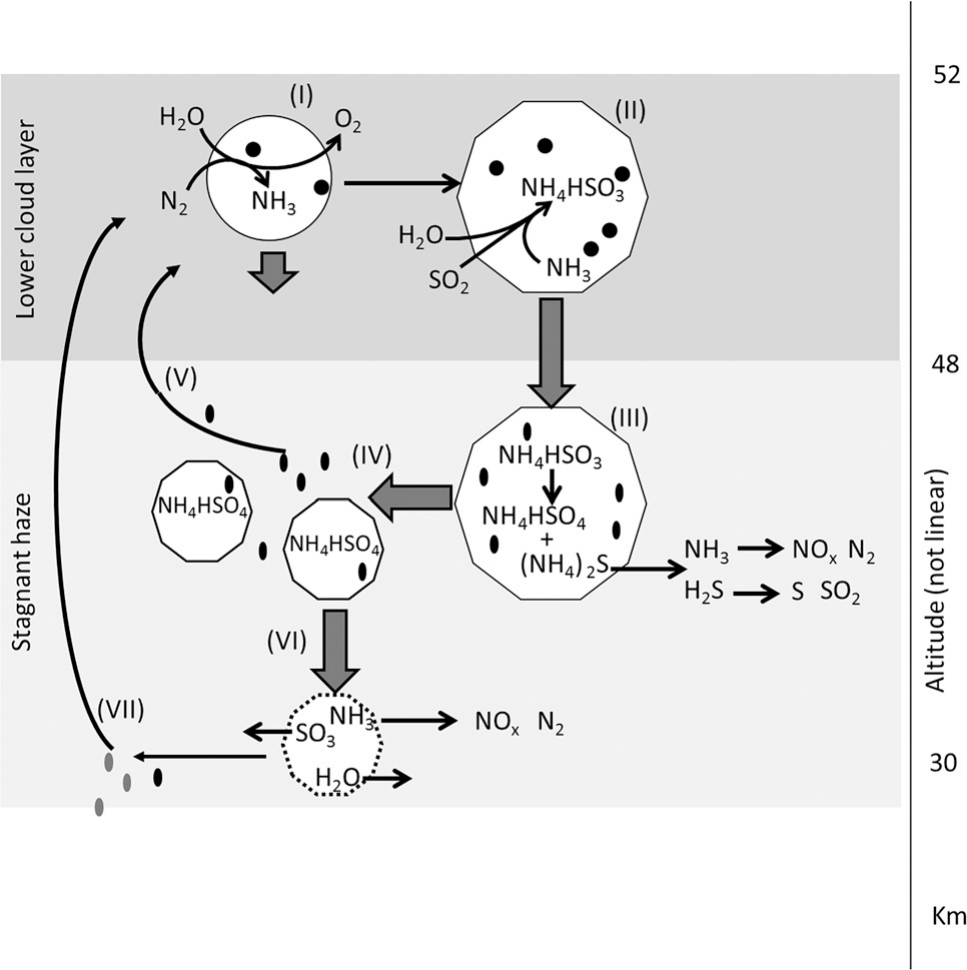
Ammonia cycle in the atmosphere of Venus. See *SI Appendix*, section 10 for details. I: NH_3_ is produced locally in the clouds from atmospheric N_2_ and H_2_O (Table 1) by metabolically active microorganisms (black dots) inhabiting cloud droplets (white circle). II: The production of NH_3_ in the droplet raises the droplet pH to −1 to 1 (from −11 on the Hammett acidity scale) by trapping the SO_2_ and H_2_O in the droplet as ammonium hydrogen sulfite (NH_4_HSO_3_). The production of sulfite salts in the droplet leads to the formation of a large, semisolid (and hence nonspherical) Mode 3 particle (white decagon). III: The Mode 3 particle settles out of the clouds where ammonium sulfite disproportionates to ammonium sulfate and ammonium sulfide; the latter decomposes to H_2_S and NH_3_, which, in turn, undergo photochemical reactions to a variety of products. IV: Disproportionation and gas release break up the Mode 3 particles into smaller haze particles and microorganism spores (black ovals), some of which return to the cloud layer (V). VI: The ammonium sulfate particles fall farther below the cloud decks, where ammonium sulfate decomposes to SO_3_, NH_3_, and H_2_O. VII: Spores released at this stage may be unviable (gray ovals), but any surviving could also be eventually transported back to the clouds.

Our model for the production of NH_3_ by life is summarized in Fig. 2.

### Toward a Resolution of Venus Atmospheric Anomalies.

**Fig. 3.**
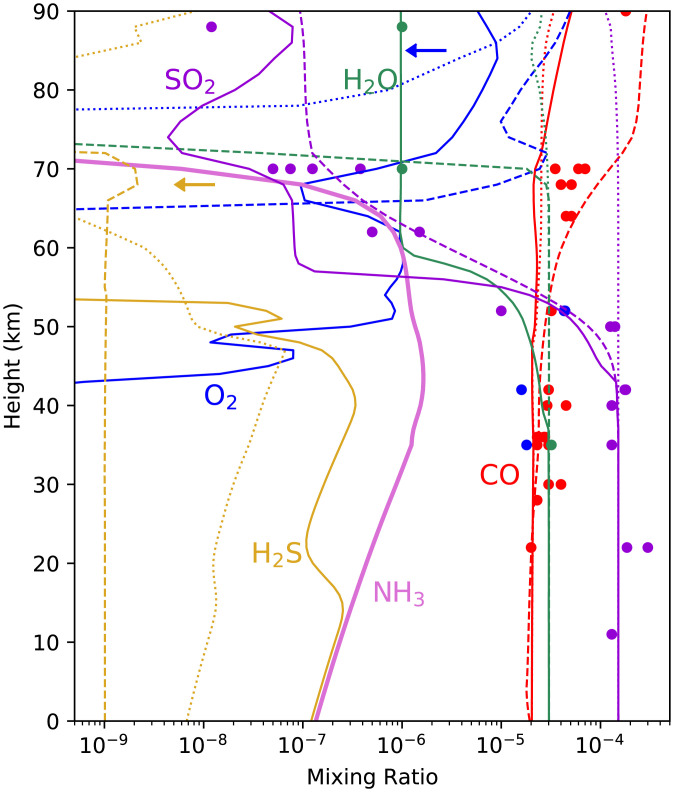
Venus atmosphere abundance profiles of key molecular species. The *x* axis is the gas fraction by volume, called the mixing ratio. The *y* axis is altitude above the surface in kilometers. The lines are gas mixing ratios from our models: with NH_3_ chemistry (solid lines), without NH_3_ chemistry (dotted lines; model in ref. 20), and without NH_3_ but with an arbitrary removal rate for SO_2_ in the cloud layers tuned to fit the data (dashed lines; model in refs. 20 and 34). The colored circles show a representative subset of collated remote and in situ data (error bars not shown) from refs. 20 (their table 4) and 33 (their supplementary table S3). Key is that the baseline model predicts no NH_3_ or H_2_S above the 1-ppb level. Models with NH_3_ chemistry have very different H_2_O, SO_2_, O_2_, and H_2_S values at some altitudes than models without NH_3_ chemistry, and improve the match to observational data. The main takeaway is that the model without NH_3_ and without the SO_2_ arbitrary removal rate (dotted line) fits the cloud layer data very poorly, whereas the model with NH_3_ (with no arbitrary constraints; solid line) fits the data much better. The boundary conditions for surface abundance in the photochemical model are listed in *SI Appendix*, Table S6.

**Fig. 4.**
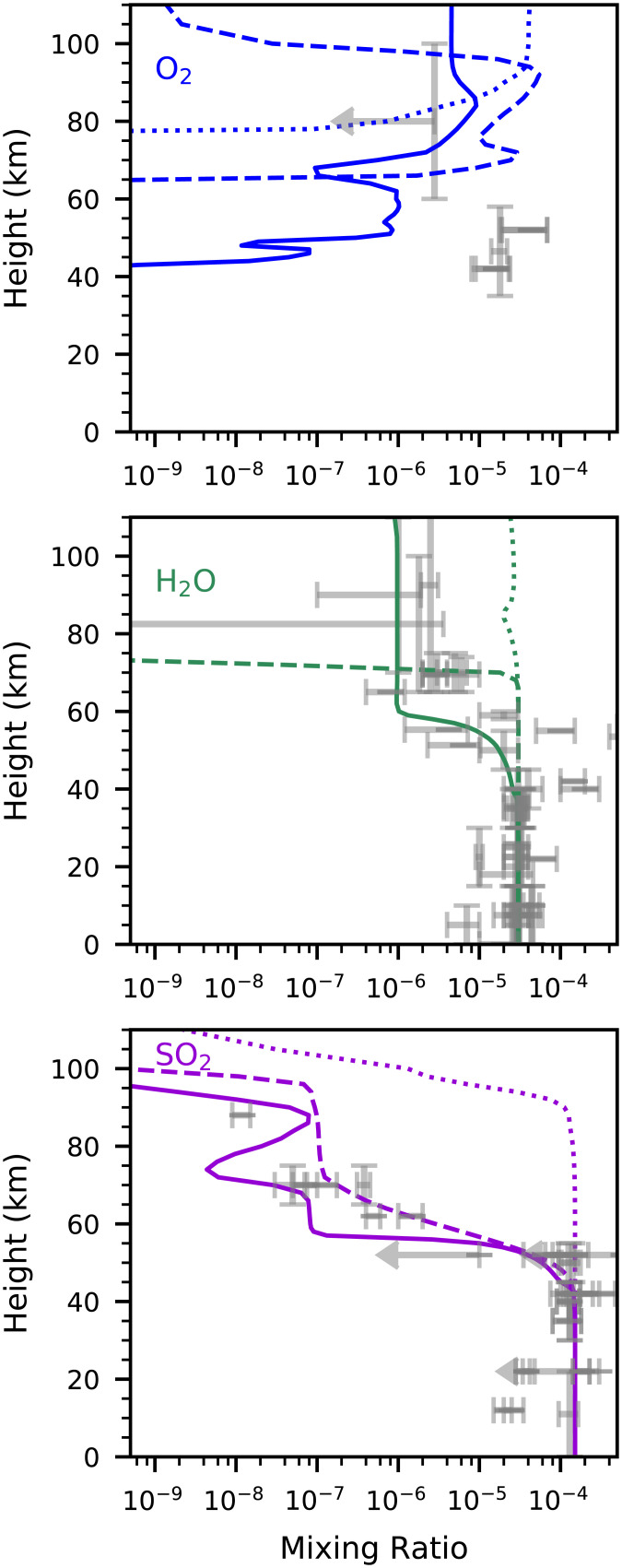
Venus atmosphere abundance profiles of three molecular species. The *x* axis is the gas fraction by volume, called the mixing ratio. The *y* axis is altitude above the surface in kilometers. The lines are gas mixing ratios from our models: with NH_3_ chemistry (solid lines), without NH_3_ chemistry (dotted lines; model in ref. 32), and without NH_3_ but with an arbitrary removal rate for SO_2_ in the cloud layers tuned to fit the data (dashed lines; model in refs. 20 and 34). Gray points with error bars are data from observations tabulated in ref. 20. (*Top*) O_2_. Our model with NH_3_ chemistry improves upon both the long-standing problem of presence and overabundance of O_2_ in the upper atmosphere and the presence of O_2_ in the cloud layers. (*Middle*) H_2_O. Our model with NH_3_ chemistry supports the presence of water vapor above the cloud layer (>80 km). (*Bottom*) SO_2_. Our models with NH_3_ chemistry (solid line) and without NH_3_ chemistry but with arbitrary constraints on SO_2_ (dashed line) both provide a fit to observed values throughout the atmosphere except for the top (>85 km). Key is that the model without NH_3_ and without the SO_2_ arbitrary removal rate (dotted line) fits the cloud layer data very poorly, whereas the model with NH_3_ (with no arbitrary constraints; solid line) fits the data much better.

The incorporation of NH_3_ in our photochemistry model of the Venusian atmosphere produces profiles of atmospheric gases that match the observed abundances of some atmospheric gases better than existing models of Venus’s atmosphere. Although NH_3_ is an input to our model, no existing Venus photochemical models include NH_3_ (e.g., refs. 22 and 43). In Figs. 3 and 4, we show a summary of the output of the modeling with NH_3_ included, compared to the same model run without NH_3_ and O_2_ input, the latter as reported in ref. 20. The atmospheric photochemistry of the clouds was modeled as described in refs. 20 and 34, and is summarized in *Materials and Methods*. Specifically, our model better explains, compared to previous models, 1) the observed disequilibria in the clouds of Venus; 2) the measured, but subsequently ignored, abundances of O_2_ in the clouds; 3) the abundance profile of water vapor; 4) the tentative detections of NH_3_ by Venera 8 and Pioneer Venus probes; and 5) the abundance profile of SO_2_ through the cloud layers. To demonstrate how well our model with NH_3_ fits the measured data, we show three model results in Figs. 3 and 4: one model with NH_3_, one model without NH_3_ but with an unphysical arbitrary depletion rate of SO_2_ (a fix common among other models in order to fit the data), and one model without NH_3_ and without any artificial chemical constraints.

We now turn to each relevant atmosphere anomaly, first reviewing the data, and then how the presence of NH_3_ helps resolve the anomaly.

#### O_2_ in the clouds is a natural outcome of NH_3_ production.

Our model provides an explanation for the presence of O_2_ in the Venus cloud layers. O_2_ has been measured via in situ measurements (44, 45). The Pioneer Venus gas chromatography (GC) reported 43.6 ppm molecular oxygen (O_2_) in the clouds at 51.6 km, 16 ppm below the clouds at 41.7 km, and no detection of oxygen at 21.6 km (23). The Venera 14 GC detected 18 ppm O_2_ average between 35 and 58 km (24). [The Large Neutral Mass Spectrometer (LNMS) on Pioneer Venus showed a signal of 32 amu, but this was attributed to O_2_ ions formed from reaction of CO_2_ in the mass spectrometer (46), and therefore was considered unreliable. However, we emphasize that this uncertainty about the source of O_2_ is specific to mass spectrometry (47).] We also note that several ground-based observations attempted to provide upper limits for the abundance of O_2_ above the clouds (48, 49). The spectroscopic searches for O_2_ have been subjected to varying interpretations (16, 17) and are claimed to be difficult to reconcile with the in-cloud O_2_ abundance detected by both Pioneer Venus and Venera probes, because one expects to observe a gradient of O_2_ from above to below the clouds. Such discrepancies can only ultimately be resolved by new in situ measurements of O_2_ in the clouds of Venus.

In the past, the validity of O_2_ has been challenged based on thermodynamics. Initial studies of the atmosphere of Venus in the 1970s and 1980s assumed the atmosphere was at thermodynamic equilibrium. One author discounted O_2_ as follows (44): “We therefore conclude, that either we have to accept a strong disequilibrium state among CO, SO_2_, O_2_ and H_2_O in the lower atmosphere of Venus, or discard at least one of the measurements in order to save the assumption of thermodynamic equilibrium. The latter course is our preferred one.” Some subsequent studies followed this argument (17, 23, 36), although not all (50), and the author himself modified his opinion in a subsequent paper (45). By now, it has been accepted for over two decades that the atmosphere of Venus is not at thermodynamic equilibrium (25, 26, 51, 52), although Venus’s atmosphere is not as far from disequilibrium as Earth’s atmosphere is (51, 52). Recently, the reanalysis of the Pioneer Venus data showed the atmosphere was farther from equilibrium than previously thought, due the presence of a range of reduced gases (27). Still, the cause of the Venus atmosphere thermodynamic disequilibrium is one of the unsolved problems in Venus science (17).

If the chemistry of NH_3_ production is the source of O_2_, then our model predicts on order 1 ppm O_2_ in the cloud level between about 50 and 60 km; 1 ppm is 20-fold lower than the measured values (23, 24). However, the value of 1 ppm at lower altitudes is far greater (15 orders of magnitude) than predicted by our and other photochemistry models that exclude NH_3_. While there are no known nonbiological processes that could produce O_2_ locally in the clouds of Venus, we note, for future work, that other biological processes such as oxygenic photosynthesis could also be contributing to the overall O_2_ budget in the clouds.

It has been suggested that O_2_ could also be produced by lightning, which is consistent with O_2_’s presence in and below but not above the clouds (53). Lightning and coronal discharge can produce O_2_ in a CO_2_ + N_2_ atmosphere (54). A thermodynamic-based calculation suggests that the amount of O_2_ possibly produced by lightning is four to five orders of magnitude too low to explain the observations (*SI Appendix*, section 8.1 and Table S4). However, the efficiency of the production of O_2_ by lightning could be tested experimentally on Earth. It is possible that all the O_2_ detections summarized above were made as spacecraft fell through high-intensity storm regions (55), but it seems an unlikely coincidence for two or three separate probes to experience storms. In addition, any NH_3_ present in the clouds would be destroyed by the lightning, and only trace amounts would reform (*SI Appendix*, section 7.1). The thermal decomposition of H_2_SO_4_ to O_2_ and SO_2_ has been suggested as an industrial process (56), but it is unlikely under Venus conditions (*SI Appendix*, section 8.2 and Fig. S5).

At altitudes above the cloud level (∼62 km), no O_2_ has been detected, strongly suggesting a fractional abundance of less than 10^−7^ (48). Yet, all existing photochemical models predict significant molecular oxygen above the clouds (e.g., ref. 22) due to the instability of CO_2_ to photolysis. CO_2_ is dissociated into CO and O, which cannot rapidly recombine because the recombination reaction is spin forbidden. Some alternative pathway, involving, for example, OH chemistry, sulfur chemistry, or chlorine chemistry, is required to restore CO_2_ (see ref. 18), but none of these pathways are sufficient to draw above-cloud O_2_ below 1 ppm (22). This mismatch between the extremely low observed O_2_ levels above the clouds and the higher predicted levels is a well-known conundrum of Venus’s cloud layer chemistry. Our model provides a partial solution by predicting a reduced O_2_ level above the clouds compared to the same model without NH_3_ (Figs. 3 and 4).

#### Model output H_2_O and SO_2_ abundance profiles are consistent with observations.

Our photochemistry model with NH_3_ production is, together with the model it is based on (20), consistent with the observed H_2_O and SO_2_ abundance profiles in and above the clouds.

SO_2_ and H_2_O have been observed on many occasions by remote campaigns, orbiters, and in situ probes (reviewed in refs. 20 and 26). For example, the Visible and Infrared Thermal Imaging Spectrometer instrument on board Venus Express observed a mean abundance of H_2_O and SO_2_, below the clouds at 30 km to 40 km, to be ∼30 ppm and ∼150 ppm, respectively (57). The observed abundances of H_2_O and SO_2_ just below the clouds are consistent between remote, orbiter, and in situ observations (20). Recall, that the 5× excess SO_2_ over H_2_O should strip all the water out of the cloud layer, and hence remove all water above the clouds as well, a solution that is not consistent with observations. The Rimmer et al. (20) model uses cloud chemistry (NH_3_ or mineral bases) to strip the SO_2_ in the clouds. As a result, water remains in the clouds and above the clouds, which agrees with the remote, orbiter, and in situ observations of a few parts per million of H_2_O above the cloud layers (reviewed in ref. 20).

Within the Venus cloud layers, there is substantial difference among measurements of water abundance in the clouds as summarized by ref. 20, which may represent varied local conditions.

In models previous to the one described here, water vapor is removed at the cloud tops by reaction with SO_3_ to form sulfuric acid, which then condenses out to form the cloud droplets. Since there is more below-cloud SO_2_ than H_2_O, all the H_2_O above the clouds is removed, some models even showing complete depletion of H_2_O (33). Yet, this depleted H_2_O above the clouds does not match observations which show plenty of water vapor above the clouds (Figs. 3 and 4). Models previous to ours solve this problem with physically unrealistic numerical fixes, either including excess H_2_O below the clouds (21) or fixing the H_2_O abundance to observed values such that any reactions involving H_2_O do not consume any H_2_O (22). Most models avoid the water vapor abundance problem altogether by restricting the calculations only to a section of the atmosphere, above the clouds or below the clouds.

Critically important is that our model without NH_3_ (33) must, similarly to other models, impose nonphysical constraints on SO_2_ chemistry in order to make the SO_2_ gas abundance profile fit observations, specifically by adding an arbitrary removal rate for SO_2_ in the clouds tuned to fit the data.

We emphasize that our model that includes NH_3_ or another base (20) is the only model known that avoids artificial fixes of SO_2_ and H_2_O. To further emphasize this point, Figs. 3 and 4 include our very poorly fitting model gas abundance profiles without NH_3_ and without the artificial SO_2_ removal rate.

Below the clouds, our photochemical model with NH_3_ predicts the same H_2_O abundance as models without NH_3_, including previous models (e.g., ref. 43).

#### NH_3_ in the clouds and below the cloud layers is consistent with tentative observations.

NH_3_ is a necessary input for our photochemistry model; indeed, the input of NH_3_ is the core assumption of our hypothesis. We therefore discuss the tentative observations of NH_3_ on Venus.

The Venera 8 descent probe reported the presence of NH_3_ in the lower atmosphere of Venus. The estimated amounts from the signal are large and varied from 0.01 to 0.1%. (For further discussion on the validity of the Venera 8 NH_3_ detection, see *SI Appendix*, section 6.) A recent reassessment of the Pioneer Venus LNMS has also provided suggestive evidence for the presence of NH_3_ and its oxidation products in gas phase in the cloud decks of Venus (27).

The Venera 8 observations were largely discounted at the time because NH_3_ is not likely to be present if Venus’s atmosphere is in thermodynamic equilibrium (36). At least one author supported the plausibility of the presence of NH_3_ in the cloud layers: Florensky et al. (50), in the late 1970s, argued that the upper parts of the Venus troposphere do not necessarily have to be in chemical equilibrium and could contain a number of minor chemical species, including NH_3_ (45).

An additional argument against the plausibility of NH_3_ is that an atmosphere containing sulfuric acid droplets cannot contain a significant amount of a free base; all of the base, in this case NH_3,_ would be sequestered in the droplets as ammonium ions. However, if the clouds have a pH of >0 and contain significant ammonium salts, then partial pressures of >1 ppm of free ammonia gas are expected over those droplets in the lower clouds (*Materials and Methods* and *SI Appendix*, section 5).

Our model provides a mechanism for the release of NH_3_ below the clouds. As the droplets gravitationally settle out of the atmosphere to higher temperatures, the droplet evaporates, and NH_3_ is released through the thermal decomposition of ammonium sulfate and ammonium sulfite. NH_3_ is subsequently oxidized to NO_x_ and N_2_ (Fig. 2). We note that a NO_x_ signal has been identified in the Pioneer Venus LNMS reanalyzed data (27).

#### Mode 3 cloud particles.

Measurements by the Pioneer Venus and Venera Probes indicate that the Mode 3 particles might not be spherical, and that their composition differs from pure concentrated sulfuric acid. (See *SI Appendix*, section 3 for a brief discussion of the observational support for nonspherical particles.)

If NH_3_ is the main neutralizing agent of the sulfuric acid cloud droplets, then the Mode 3 cloud particles in the lower clouds must be supersaturated in ammonium salts, with a small liquid phase, and therefore are not liquid droplets of concentrated sulfuric acid. Thus, the mechanism proposed here predicts that the Mode 3 particles in the lower cloud are solid or semisolid, and hence likely to be nonspherical.

Specifically, the Mode 3 (largest) cloud particles in the lower cloud must be 9.3 molar to 18.1 molar in ammonium salts in order to provide sufficient downward transport of SO_2_ to produce the observed drop in SO_2_ concentration across the clouds (*Materials and Methods* and *SI Appendix*, section 5). Such concentrations are not implausible if the Mode 3 particles in the cloud are actually a semisolid slurry of ammonium salts and sulfuric acid.

We note that presence of NH_3_ creating nonspherical Mode 3 particles is consistent with the Mode 1 and/or Mode 2 particles being of quite different composition than the Mode 3 particles. If NH_3_ production were the result of biological activity, then life could be confined to the larger Mode 3 particles, which have more volume. If NH_3_ was produced by a nonbiological process, then it would be expected to apply to particles of all sizes, and not discriminate in favor of Mode 3 particles. However, the data on particle size and shape is consistent with Mode 1 and 2 particles being spherical (29).

Our model also explains the presence of the so-called stagnant haze layer below the cloud decks (30 km to 47 km altitude) (9). If the large Mode 3 particles are made of mostly solid ammonium sulfite and ammonium sulfate, then evaporation of any residual H_2_SO_4_ at the cloud base leaves dry solid particles. The subsequent thermal disproportionation of the remaining salts generates gas that shatters the particles at the cloud base (∼100 °C at ∼47 km), and the fragmented particles form the haze. The haze that settles down and is not mixed back up into the clouds decomposes at ∼200 °C at the bottom of the stagnant haze layer at ∼30 km (Fig. 2). The layered structure and the altitudes of the boundaries between the layers is therefore a natural consequence of the ammonia-based cloud chemistry. See also ref. 7 for a discussion of the composition of the haze layer.

#### H_2_S below the clouds.

We also note that our model predicts the presence of H_2_S below the clouds (Figs. 2 and 3). The presence of H_2_S is consistent with the tentative detection of H_2_S below the clouds by the Venera 14 GC (24), which is the only in situ measured abundance value for H_2_S. If NH_3_ is present in the Venus atmosphere, H_2_S is a result of disproportionation of NH_4_HSO_3_ that yields NH_3_, H_2_S, and H_2_O to the atmosphere below the clouds, and hence is a unique output of our model. H_2_S was also tentatively identified in the recent reanalysis of the Pioneer Venus LNMS data (27). H_2_S, however, is a known volcanic gas on Earth so it is likely produced by volcanoes on Venus as well.

## Discussion

Our model provides a view of the habitability of Venusian clouds. Concentrated sulfuric acid would make the Venusian cloud environment both chemically aggressive and extremely dry (7, 14). Our model removes the issue of extreme acidity for a subset of cloud particles from consideration.

Our model implies that the Mode 3 cloud particles cannot be all composed of concentrated H_2_SO_4_. Instead, there has to be a population of cloud particles that are less acidic and have a higher pH (between −1 and 1) than concentrated sulfuric acid. Specifically, our model predicts that the Mode 3 cloud particles are semisolid ammonium sulfites and sulfates (Fig. 2) with a pH as high as one (Fig. 1). We emphasize that not all droplets need to contain semisolid ammonium sulfite and (if the NH_3_ is made by life) ammonia-producing microorganisms.

Relevant to the Mode 3 cloud particles is a recent, independent finding that the Mode 3 cloud particle composition is not primarily sulfuric acid, but instead is consistent with some particles being ammonium hydrogen sulfate (NH_4_HSO_4_), as also predicted in our analysis. Mogul et al. (58) base this finding on a reanalysis of the Pioneer Venus legacy data on the refractive index of the Venusian cloud droplets, independent of atmospheric chemistry. Also, independently from our work presented here, Mogul et al. (58) have described the potential for phototropic synthesis of NH_3_ to neutralize sulfuric acid cloud droplets, leading to the Mode 3 particle possibly containing NH_4_HSO_4_.

A pH of zero to one is within the range of environments known from Earth to be habitable and, in fact, to be inhabited. Life can grow in acid (pH = 0) aqueous environments (35), and microbial growth in solutions as acidic as a pH = −0.5 has been claimed (59). Furthermore, most of the Mode 3 particles have been detected at altitudes in the temperature range (60 °C to 80 °C), a range that overlaps with environments known to harbor thermophilic acidophiles on Earth (with life that can grow in temperatures up to 100 °C; e.g., refs. 60–63).

Remarkably, examples of life on Earth secreting NH_3_ to neutralize a droplet-sized acidic environment exist. Pathogens such as *Mycobacterium tuberculosis* and *Candida albicans* can neutralize the interior of phagosomes (acid-containing vesicles inside cells used for digestion of captured organic material) by secreting ammonia, thus evading destruction (64–66). Some plant pathogens also secrete ammonia to neutralize local pH in their target plant cells (67). By contrast, pond-dwelling acidophilic microorganisms adapt to low pH in other ways, because it is implausible for them to neutralize an entire river or pond.

Challenges to life in the Venus atmosphere remain. The extreme aridity of the Venus cloud environment has been well known for decades (e.g., ref. 68), having been often described (e.g., refs. 7, 14, and 34), and most recently reviewed in ref. 69, and remains a significant challenge to life as we know it. Our model predicts a water vapor abundance mixing ratio of 10^−5^ in the lower clouds, that is, a relative humidity of 0.02% (depending on temperature). This is ∼50-fold lower than the lowest water activity known to support life on Earth. (We note that terrestrial life can survive extremely hot and dry environments as spores or other inactive forms, as summarized in the legend to Fig. 2 and *SI Appendix*, section 10, but these are not actively growing, and to survive an ecosystem requires at least some cells or organisms to be actively growing.) The range of in-cloud water vapor abundance mixing ratios reported in the literature is very large (5 ppm to 0.2%), as summarized by ref. 20, which may represent the presence of more clement local conditions. All global models may therefore represent an average of extremely arid “desert” regions and much more humid “habitable” regions.

The extreme aridity is a reflection of the very low number density of hydrogen atoms in the Venusian atmosphere. The scarcity of H atoms argues against the presence of life. Terrestrial biochemicals are typically ∼50% hydrogen by atom number (as illustrated by the database of natural products compiled by ref. 70; *SI Appendix*, section 9 and Table S5). However, much of the water in a bacterial cell is derived from reactions of the metabolites within the cell (71–73). For example, under active growth of *Escherichia coli*, up to 70% of the intracellular water is generated during metabolism and not transported across the membrane from the outside environment (71). If there is life on Venus, it is therefore likely to have substantially different biochemistry from Earth’s, and, if it is based on water as a solvent, it is likely to have very different strategies for water accumulation and retention to combat extreme aridity of the clouds. We note, however, that the lack of hydrogen is not just a challenge for the habitability of Venus’s clouds but also a challenge for making detectable amounts of any hydrogen-saturated gas-phase species, such as NH_3_, by any mechanism, abiotic or biological.

We note that additional challenges such as nutrient scarcity or high energy requirements are comparatively less limiting than aridity; for an in-depth discussion of the challenges to life in the Venusian clouds, see ref. 7.

An origin for life on Venus is an open question. If life exists in the Venus clouds, it may have originated on the Venus surface and migrated into the clouds. One model of Venus’s evolution to its modern state suggests that Venus had clement surface conditions after formation, only to have entered the current greenhouse runaway after up to 3.5 billion years (74, 75). This model is dependent on a range of specific conditions but, if correct, suggests that Venus in the past had similar conditions to those under which life originated on Earth. If life emerged on the surface, terrestrial precedent suggests that some organisms would adapt to living some of the time in the clouds (reviewed in ref. 7). The microbial acid-neutralizing strategy provides a facile evolutionary path to Venusian cloud life. As the Venus surface became increasingly hot and uninhabitable, cloud dwelling would become a permanent lifestyle.

As the atmospheric chemistry changed to high acidity, the cloud-dwelling organisms would adapt by neutralizing their droplet habitats. A plausible evolutionary path is therefore suggested by the unique role of a droplet environment in the acid-neutralizing strategy, and the proposed history of Venus. We note, however, that, if life is the source of NH_3_ on Venus, it very likely does not resemble the elemental ratios of life on Earth and likely has a different biochemistry than life on our planet, specifically adapted to the unique challenges of the Venusian cloud environment.

The Venus low D/H ratio (76) and the possible existence of felsic rocks which form in the presence of water (77–80) imply the presence of past Venus oceans, yet the debate on whether or not Venus ever had oceans continues. Recently, Turbet et al. (81) demonstrated, with a three-dimensional (3D) global climate model, that Venus may have been too hot early on for water oceans to form. Their climate model shows that the steam atmosphere of early Venus never condensed on the planet’s surface to form liquid water oceans. Instead, according to the model, water vapor condensed on the nightside of the planet to form clouds that warmed the surface by absorbing and reemitting the planet’s outgoing infrared radiation (81). However, Turbet et al. (81) do state that a comprehensive sensitivity study is needed to quantitatively confirm their result, as cloud and atmospheric circulation feedbacks can vary nonlinearly and nonmonotonically with rotation period. The newly selected VERITAS and EnVision missions, as well as DAVINCI’s instruments, should solidify or rule out the possibility of the past water-rich era of Venus, by a combination of D/H measurements and multispectral imaging of the tesserae regions for mineral compositions.

## Summary and Critical Future Measurements

Our hypothesis of locally produced NH_3_ in the Venus clouds explains a number of anomalies in the atmosphere and clouds of Venus. Our photochemical model of the consequences of NH_3_ production explains the SO_2_ depletion in the clouds and vertical abundance profile of H_2_O, building on the work of ref. 20, explains the presence of O_2_ in the clouds, supports the in situ detection of H_2_S below the clouds, and explains the nonspherical nature of Mode 3 particles. While the presence of other mineral bases could contribute, none of them can explain the parts per million levels of O_2_ in the clouds or the tentative presence of NH_3_. No definitive source for NH_3_ has been identified; in chemical terms, biological production is the most plausible, but the concept of life in the clouds of Venus remains controversial. Many of the in situ observations should be repeated for confirmation, and more model work is needed to fully resolve the vertical abundance profiles of relevant gases.

We must be careful not to fall for a conjunction fallacy. While life may explain the combined anomalies with some external assumptions, there may yet be a chemical explanation for each individual anomaly.

An in situ Venus probe can support or refute our proposed view of Venus as an inhabited planet with the following measurements.

### Gases.

• Establish the existence of NH_3_ and O_2_ in the cloud layers.
• Measure the amounts of NO_x_ to establish which NH_3_-destruction pathway dominates.
• Determine the specific altitude-dependent abundance profiles of H_2_O, SO_2_, and H_2_S, ideally with day and night measurements to inform chemistry sources and sinks.

### Cloud Particles.

• Confirm the nonspherical, semisolid nature of Mode 3 cloud particles and identify them as ammonia salts.
• Measure the pH of cloud particles, especially Mode 3 cloud particles
• Detect organic molecules in cloud particles; if found exclusively in the larger particles, this would be an indicator of life.

### Search for Life.

• Analyze a large number of individual cloud particles, especially Mode 3, for morphological and chemical signs of life.

In the meantime, a public release of original data from the Russian Venera and Vega missions could enable further support or refutation of current models and predictions, and would provide needed context for future mission results.

We have presented an initial analysis of several sources for the NH_3_ on Venus. We have argued that biological production may be a potential source of both NH_3_ and O_2_ that we have identified that meets the quantitative requirements for NH_3_ production. Although the biomass required to make NH_3_ and O_2_ at the required rate is not unrealistic, at 0.05% of the total biomass on Earth and ∼1.5% of the total Venusian cloud mass, life in the clouds of Venus has been considered implausible because of very high acidity, very low water activity, and scarcity of hydrogen atoms. By predicting a Mode 3 particle pH of −1 to 1 due to neutralizing NH_3_, our work implies both that Venus clouds are more habitable than previously thought and, by the requirement of locally produced NH_3_, that clouds may be inhabited. We hope our work will encourage further studies into habitability and astrobiological potential of Venusian clouds.

## Materials and Methods

### Photochemical Model.

The details of the model are provided in *SI Appendix*, section 4. In summary, we employ a 1D Lagrangian photochemistry/diffusion code that follows a single parcel as it moves from the bottom to the top of the atmosphere. The temperature, pressure, and actinic UV flux are prescribed at each altitude in the atmosphere (20).

### Calculation of Flux of Ammonia.

We calculate the flux of ammonia necessary to maintain the observed gradient of SO_2_ through the clouds following the method of Rimmer et al. (20). The goal is to explain the removal of nearly all of 3.5·10^15^ cm^−3^ of SO_2_ (1.5·10^−4^ bar at 300-K level of the atmosphere) that should be present from upward mixing from volcanic sources and recycled SO_2_. The time taken for SO_2_ to mix through the region 45 km to 65 km is calculated using Lee et al.’s (82) equation 7,

$$\text{Time}\approx \;2\cdot \frac{{\text{H}}^{0}.\text{δh}}{{\text{K}}_{\text{zz}}}\;=2.6\cdot 10^{8}\;\text{s}\approx \;8.25\;\text{Earth}\;\text{years}.$$

In other words, SO_2_ will be replenished in the atmospheric cloud layers in 8.25 Earth years, and this is the timescale that the presence of NH_3_ needs to remove SO_2_. The atmospheric scale height H_0_ ≈ 6.5 × 10^5^ cm is the average scale height in the atmospheric cloud layers, δh = 2 × 10^6^ cm is the distance between 45 and 65 km, and K_zz_ = 10^4^ cm^2^⋅s^−1^ is the eddy diffusion coefficient throughout the atmospheric cloud layers. The flux (per square centimeter per second) of SO_2_ into the clouds is therefore given by

$$\text{Φ}=\;\frac{\text{Amount}\cdot \text{Distance}\;}{\text{Time}}=\;\frac{\left(3.5\cdot 10^{15}{\text{cm}}^{-3}\right)\cdot (2\cdot 10^{6}\;\text{cm})}{2.6\cdot 10^{8}\;\text{s}}=2.7\;\times \;10^{13}\;{\text{cm}}^{-2}\cdot {\text{s}}^{-1}.$$

Recall that there is a one-to-one molar ratio for NH_3_ to remove SO_2_. Given the mass of NH_3_, is the flux rate above is equivalent of 1.1 × 10^11^ tonnes per year of NH_3_.

### Calculation of Concentration of NH_3_ in Particles.

The concentration of salts in the cloud droplets can be estimated from the concentration necessary to provide the flux of NH_3_ as calculated above. The necessary flux of NH_3_ is dependent on the size of the particles, and hence the particles’ rate of fall. For a given particle size, we can calculate the rate of fall, and hence the volume of cloud material removed per second, and, from this, the concentration of salts in that volume needed to provide the flux calculated above. See *SI Appendix*, section 2 for the details on the calculation of the concentration of ammonium salts in the lower cloud particles.

### Calculation of Concentration of Gaseous NH_3_ over Droplets.

The concentration of gaseous NH_3_ over an acid droplet containing dissolved NH_4_^+^ was calculated as follows. The fraction of total N species that is present as NH_3_ and as NH_4_^+^ can be calculated from the acid dissociation constant (pK_a_) of NH_3_ as the pH. The concentration of NH_3_ over solution can be calculated from the solution concentration and Henry’s constant (*SI Appendix*, section 5 and Fig. S3). Both pK_a_ and Henry’s constant are dependent on temperature.

## Supplementary Material

Supplementary File

## Data Availability

Previously published data were used for this work (20). All other study data are included in the article and/or *SI Appendix*.

## Change History

January 21, 2022: The byline has been updated.
