# Supplementary material for: Production of ammonia makes Venusian clouds habitable and explains observed cloud-level chemical anomalies
Source: Proc Natl Acad Sci U S A. 2021 Dec 20;118(52):e2110889118. doi: 10.1073/pnas.2110889118 (PMC8719887; doi:10.1073/pnas.2110889118)
Supplement: Supplementary File [file pnas.2110889118.sapp.pdf]

PNAS
www.pnas.org

Supplementary Information for
Production of Ammonia Makes Venusian Clouds Habitable and Explains Observed Cloud-Level Chemical Anomalies

William Bains[1,2], Janusz J. Petkowski[1], Paul B. Rimmer[3,4,5], Sara Seager[1,6,7,*]

[1]Department of Earth, Atmospheric, and Planetary Sciences, Massachusetts Institute of Technology, Cambridge, MA 02139, USA
[2]School of Physics & Astronomy, Cardiff University, 4 The Parade, Cardiff CF24 3AA, UK
[3]Department of Earth Sciences, University of Cambridge, Downing St, Cambridge CB2 3EQ, United Kingdom
[4]Cavendish Laboratory, University of Cambridge, JJ Thomson Ave, Cambridge CB3 0HE, United Kingdom
[5]MRC Laboratory of Molecular Biology, Francis Crick Ave, Cambridge CB2 0QH, United Kingdom
[6]Department of Physics, Massachusetts Institute of Technology, Cambridge, MA 02139, USA
[7]Department of Aeronautics and Astronautics, Massachusetts Institute of Technology, Cambridge, MA 02139, USA.

*Sara Seager

Email: seager@mit.edu

This PDF file includes:

- Supplementary text
- Figures S1 to S5
- Tables S1 to S6
- SI References

Supplementary Information Text

1. Ammonia is the only plausible base that can be made locally in clouds.

A base is required that can play the role of B in the reaction:

$SO_2 + H_2O + B \rightarrow SO_2 + BH^+ + OH^- \rightarrow BH^+ + HSO_3^-$.

The pKa of the buffer H_2SO_3/HSO_3^- is 1.71, so we require a base for which the pKa of the pair B + H^+ / BH^+ is greater than 1.71, to that there is a pH (H^+ concentration) at which HSO_3^- is favored over H_2SO_3 and BH^+ is favored over B. If the base is to be formed locally in the clouds and not transported from the surface, then it must be formed from locally available elements. Of the possible bases that could be formed from the elements present in volatile compounds in the clouds (H, C, N, O, F, P, S, Cl), only ammonia (NH_3) or hydrazine fulfil that criterion as described in Table S1.

2. Calculation of NH_4^+ salt concentration in cloud droplets.

The rate of settling of cloud particles can be estimated as follows. The lower cloud is characterized by particles in three size modes (1, 2). These particles are assumed to be roughly spherical. Assuming that they are small enough to fall under a laminar flow (Stokes) regime, then their settling velocity can be calculated from

$$v = \frac{2}{9}\cdot\frac{\rho}{\mu}\cdot g \cdot r^2.100 \quad \text{cm/sec,} \qquad (1)$$

where v = terminal velocity in cm/sec, ρ = droplet density in kg/m^3 (assuming that this is very much greater than the gas density, so the gas density can be neglected), μ = dynamic viscosity of the gas, g = acceleration due to gravity in m/sec^2, which on Venus is 8.87 m/sec^2, and *r* is the particle radius in meters. μ for pure CO_2 at 100 °C is ~$1.85\cdot10^{-5}$ kg/m/sec, and this is taken as the viscosity of the Venusian atmosphere. The particles are assumed to be roughly spherical – a slurry of liquid and solid is likely to adopt a roughly spherical form in free fall. The density of ammonium hydrogen sulfate is 1.79 g/cm^3, the density of sulfuric acid is 1.74 -1.82 g/cm^3, depending on concentration. We therefore adopt a density of 1.75 g/cm^3.

The rate at which material falls from the clouds is therefore

$$m_f = d \cdot v \quad \text{g/cm}^2\text{/sec,} \qquad (2)$$

where m_f is the mass falling past a 'cloud base' level, *d* is the mass density of cloud particles and v is the velocity. The mass density depends on the density of the particles, which itself depends on the concentration of solutes in H_2SO_4 (assuming that H_2SO_4 is the solvent phase). At the cloud base the droplets have previously been assumed to be ~100% sulfuric acid at ~100 °C, which has a density of 1752 kg/m^3 (3). There is no data on the density of ammonium sulfite solutions in concentrated sulfuric acid, but the density of ammonium sulfate in concentrated sulfuric acid is only slightly different from the density of acid (4).

The size distribution of the droplets is taken from (2) (although we note that their modelling of the clouds has been contested (5, 6)). The distribution of cloud particles was taken from the analysis of the Pioneer Venus sounder measurements reported in (2), for the 49 km (i.e. lower cloud) layer. They deduced a trimodal cloud distribution, with the distribution as described in Table S2.

Knollenberg and Hunten (ref. (2)) note that the log normal distribution of Mode 3 particles significantly under-estimates the abundance of the largest Mode 3 particles. We confirm this in Figure S1, top panel, where distributions have been fitted to the observed abundance data for the 49 km sample, from Figure 12 of (2). Large particles have a disproportionate influence on the rate of loss of material from the cloud by setting for two reasons; they have larger volume and hence contain more mass, and have a larger cross-sectional area and hence settle faster. We therefore adopted a log-log-normal distribution (i.e. a distribution that is normal for log(log(diameter)), a function that is not defined for diameter < 1) for Mode 3 particles, which fits the observed data better (Figure S1, bottom panel). We do not claim that this has a realistic physical interpretation, just that this interpolates the observed particle distribution better than a log-normal distribution.

This particle distribution is expected to settle at a rate yielding $6.71 \cdot 10^{-12}$ liters/cm^2/second. 97.6% of this settling is carried by Mode 3 particles. The value of the necessary flux of SO_2 from the clouds from equation (1) can now be compared to the flux of particle mass from the clouds in equation (2). Assuming a density of 1750 kg/m^2 for the droplets made mostly of sulfuric acid or its salts, the concentration of SO_2 is

$$[\mathrm{SO_2}] = \frac{\rho}{N_a} \cdot \frac{d}{m_f} = 9.34 - 18.05 \text{ molar} \qquad (3)$$

where ρ and m_f are as defined above, d is the density of the cloud droplets and N_a is Avagadro's number, $6.023 \cdot 10^{23}$, with a range of values depending on whether the mean Mode 3 particle diameter is 7.5 or 8.5 microns. A saturated solution of ammonium hydrogen sulfite in water is ~5 molar. The density of solid ammonium sulfite is 1.41g/cm^3 (7) (no density of available for solid ammonium hydrogen sulfite), which implies that solid ammonium sulfite has ~12 moles per 1000 cm^3. Thus, for settling of cloud particles to be the sole removal mechanism for ammonium sulfite, and ammonium sulfite formation to be the sole mechanism for removal of SO_2, the cloud particles must be super-saturated in ammonium salts, and may be mostly solid ammonium salts with a small liquid phase of aqueous sulfuric acid. We note that this model presents the "upper limit" of ammonium sulfite concentration needed to explain the depletion of SO_2 in the clouds, as NH_3 does not have to be the only neutralizing agent in the clouds (8).

3. Evidence that Mode 3 particles are non-spherical.

Estimates of the refractive index of the particles in the lower cloud suggest a value of ~1.33 assuming spherical droplets (9), which is lower than any plausible value for concentrated sulfuric acid. The droplets could be more dilute sulfuric acid, but this is not compatible with the vapor pressure in the clouds (10). This anomaly can be resolved if the particles absorb a small amount of incident light; however (11) find no noticeable absorption in the lower clouds. An alternative explanation is non-spherical particles (2, 9). which implies non-liquid ones. The Pioneer Venus sounder data supports non-spherical particles for the largest Mode 3 particles. The optical array spectrometer (OAS) instrument had three photodiode arrays which measured the shadow of particles as they passed, which makes the particle size measurement independent of particle composition. In the lower cloud, the range 2 array (which measures particles of 5 – 53 μm diameter) counted 72 particles larger than 16 μm, whereas the range 3 array (which measures particles of 16 – 181 μm diameter) counted only 3 particles (2). The comparison is complicated by different sampling volume and different sensitivities, but a discrepancy significant to $p<0.01$ remains. Such discrepancies are well-known in using OAS instruments to measure non-spherical particles such as snowflakes (12). The particle size distribution as determined by the OAS and reflectance as determined by the solar flux radiometer are also inconsistent with spherical particles (13).

We note that the early Venera and Vega measurements of the cloud particles' properties are consistent with data acquired by Pioneer Venus (14) and likewise have also returned inconsistent data about the cloud composition. We acknowledge however that the Mode distribution of the

Venus' cloud particles is a topic of decades-long heated debate. Several studies questioned the existence of the large Mode 3 particles altogether, e.g. (6, 15), and for example claimed that Mode 3 could in fact be a large "tail" of the liquid Mode 2' distribution, once calibration errors are taken into account (5).

Such decades-long lingering questions on the true nature of the Venus cloud particles strengthen the need for a renewed campaign of in situ measurements to characterize the aerosols.

4. Details of atmospheric photochemistry model.

In the rest-frame of the parcel, diffusion terms are accounted for by time-dependence of the chemical production, P_i (cm^3 s^{-1}), and loss, L_i (s^{-1}), and so below the homopause, resulting in the equation:

$$\frac{\partial n_i}{\partial t} = P_i[t(z, v_v)] - L_i[t(z, v_z)]n_i, \quad (4)$$

where n_i (cm^{-3}) is the number density of species *i*, *t* (s) is time, *z* [cm] is atmospheric height. Convergence conditions and further details, including the chemical network, are given in (8). We implement the effective rates for SO_2 depletion into the clouds under the assumption that the NH_3 chemistry is sufficient to regulate the in-cloud SO_2 abundances. We modify the model to add in-cloud fluxes of O_2, NH_3, H_2S and SO_3. The degassing rates represent the following reactions:

$4(NH_4)_2SO_3 \rightarrow NH_3 + H_2S + 3(NH_4)_2SO_4$
$(NH_4)_2SO_4 \rightarrow 2NH_3 + H_2O + SO_3$)
$2N_{2(aq)} + 10H_2O_{(l)} \rightarrow 4NH_4^+OH^-_{(aq)} + 3O_{2(aq)}$

with the rates defined as:

Eq. (5):

$$\Phi(O_2) = \Phi_0\left\{\frac{1}{2}\tanh\left[\frac{1}{2}(z-h_4)\right]\cdot\tanh\left[\frac{1}{2}(h_6-z)\right]+\frac{1}{2}\right\}$$

Eq. (6):

$$\Phi(H_2S) = \frac{1}{2}\Phi_0\left\{\frac{1}{2}\tanh\left[\frac{1}{2}(z-h_3)\right]\cdot\tanh\left[\frac{1}{2}(h_5-z)\right]+\frac{1}{2}\right\}$$

Eq. (7):

$$\Phi(SO_3) = 3\Phi_0\left\{\frac{1}{2}\tanh\left[\frac{1}{2}(z-h_1)\right]\cdot\tanh\left[\frac{1}{2}(h_2-z)\right]+\frac{1}{2}\right\}$$

Eq. (8):

$$\Phi(NH_3) = \Phi_0\left\{\frac{1}{2}\tanh\left[\frac{1}{2}(z-h_3)\right]\cdot\tanh\left[\frac{1}{2}(h_5-z)\right]+\frac{1}{2}\right\}+3\Phi_0\left\{\frac{1}{2}\tanh\left[\frac{1}{2}(z-h_1)\right]\cdot\tanh\left[\frac{1}{2}(h2-z)\right]+\frac{1}{2}\right\}$$

where h_1 = 30 km, h_2 = 35 km h_3 = 40 km, h_4 = 45 km, h_5 = 50 km and h_6 = 65 km. The heights are set based on altitudes i) at which ammonium sulfate decomposes (h_1 and h_2), ii) at which ammonium sulfite disproportionates (between h_3 and h_5), and iii) at which oxygen is presumed to be produced in the lower cloud (between h_4 and h_6). The altitudes h_3, h_4, h_5 and h_6 are set by the temperature profile of Venus's atmosphere. $\Phi_0 = 1.5 \cdot 10^7$ cm^{-3} s^{-1} is the degassing rate needed

to account for the amount of ammonium sulfite sufficient to store the in-cloud SO_2. The leading factors are stoichiometric. The overall fluxes of our model are shown on Figure S2.

The boundary conditions for surface abundance in the photochemical model are listed in Table S6.

5. Prediction of vapor pressure of NH_3 over solution of different pH.

The abundance of NH_3 in the atmosphere over a solution of given pH was calculated as follows. The abundance of free NH_3 in a solution of ammonium salts is given by

$$pKa = \log_{10} \cdot \frac{[NH_3]\cdot[H^+]}{[NH_4^+]},$$

where [NH_3] is the concentration of free NH_3 in solution. The concentration of NH_3 in gas phase is given by

$$\left[\mathrm{NH_{3(g)}}\right] = H \cdot \left[\mathrm{NH_{3(aq)}}\right],$$

where *H* is Henry's constant. The pKa for NH_3 as a function of temperature and pH was calculated as described by (16). Henry's law as a function of temperature was taken from averaged values presented by (17). The result for different pH values the Mode 3 particles is shown in Figure S3.

6. Commentary on the Venera 8 detection of NH_3.

The Venera 8 gas analyzer had a dedicated instrument to detect NH_3 using bromophenol blue as an indicator of a basic atmospheric component (18). This experiment for the determination of the NH_3 mixing ratio in the 44 to 32 km altitude region estimated the NH_3 mixing ratio between 0.01% and 0.1%, from the color change of "bromophenol blue" (18). The Venera 8 values are difficult to reconcile with the proposed 6 ppb upper limits for NH_3 abundance above the clouds (19), unless the NH_3 loss in the upper atmosphere is balanced by a constant production that is localized to the lower atmospheric regions (the clouds and the stagnant haze layer below). Such discrepancies can only ultimately be resolved by new in situ measurements of NH_3 in the clouds of Venus.

It appears that the instrument detected absorbance change in bromophenol blue exposed to atmosphere compared to a control chamber; however absorbance was measured by photoresistor response to an (undescribed) light source (18, 20), and so might not have been able to distinguish color change due to pH change from color change resulting from indicator breakdown, as also pointed out by (21) who postulated that the Venera 8 NH_3 detector could have responded to gaseous sulfuric acid, as bromophenol blue turns violet-red in concentrated sulfuric acid.

7. Non-biological pathways of formation of NH_3 in the clouds of Venus.

7.1. Production of NH_3 by lightning.

We model the production of NH_3 by lightning following the model of (22). The molecules intercepted by a lightning bolt are assumed to be broken into their component atoms, and then these recombine at random depending solely on the relative numbers of atoms. Thus, to make NH_3, a nitrogen atom must collide with three hydrogen atoms and *not* collide with any other atom. Thus, the fraction of N atoms that form NH_3

$$f(N) = \left(\frac{H}{T}\right)^3,$$

where f(N) is the fraction of N atoms that form NH_3, H is the number of N atoms/cm^3 and T is the total number of atoms /cm^3. Assuming 100 lightning strikes/second, a ratio of cloud:cloud vs cloud:ground lightning strikes of 100:1, 100 diameter lightning bolts that are 10 km long in the clouds and 45 km long between clouds and ground, and using the range of gas concentrations used in (22), we find the production rate of NH_3 is 16.7 moles/year (standard deviation 1.26), i.e. 285.6 grams/year. An example of the calculation is given in Table S3.

The examples of the calculations presented in Table S3 assume a lightning strike rate similar to Earth. The rate would have to be ~10^{9} times the terrestrial value to approach the production rate needed to explain the presence of NH_3 in the clouds, even assuming this mechanism. Such extreme lightning activity seems implausible. This is in line with previous work on NH_3 as a biosignature gas (23).

As an extreme calculation, we calculated what the thermodynamic equilibrium concentration of NH_3 and oxygen would be if the entire atmosphere was 'shocked' to high temperature and quenched at 1200 K, the temperature at which lighting or shock-driven reactions can be considered to be quenched (Zahnle et al. 2020, their Fig. 1 (24)). The result is shown in Table S4. Such a scenario would require the entire atmosphere to be filled with lightning, not dissimilar to the scenario required in the calculation above, in Table S3.

7.2. UV photolysis producing NH_3.

We can model the photochemical production of NH_3 in two ways. The first is to perform a full photochemical model of the atmosphere, using available kinetic data. This has been done previously, and suggests negligible (~ parts per tirillion, ppt) NH_3 abundance (e.g. the Rimmer et al hydroxide cloud chemistry model achieves ~11 ppt of NH_3 (8)).

There remains the possibility that non-equilibrium photochemistry using pathways not modelled could generate NH_3. Given that the photochemistry of nitrogen species in gas phase has been explored very extensively, the most likely context for this would be the photochemistry of N_2 in sulfuric acid solution, which to our knowledge has not been explored. N_2 is not protonated in H_2SO_4; indeed, N_2 is frequently used as an inert carrier gas for investigations of reactions of gases with H_2SO_4. It also is unlikely that reaction in H_2SO_4, a powerful oxidizing agent, would produce a reduced gas. However the possibility remains, and could be explored experimentally.

7.3. Volcanic production of NH_3.

For the purposes of exposition, we assume that Venusian volcanoes produce NH_3 at the same rate as terrestrial volcanic and hydrothermal systems. This will grossly over-estimate production of NH_3, as the large majority of terrestrial NH_3 is produced as a result of the presence of abundant water in volcanic and hydrothermal systems (for example, by reaction in serpentizing systems). However, this provides as basis for comparison.

A tabulation of the fraction of gas over 100 °C emitted by terrestrial fumaroles is summarized in Figure S4. (Gas below 100°C is liable to condense and wash out any NH_3 present).

The geometric mean ratio NH_3/CO_2 was 0.004. Estimates of the global volcanic CO_2 emission is 6 – 11 x 10^{12} moles/year (25). This suggests a flux of NH_3 of a maximum of ~640 g/second. This is ~5 x 10^4 times too low a rate to fit the model presented here. For volcanism to be a plausible source of the NH_3 required by this model, a) volcanism on Venus would have to be more than 10^4 times as volcanically active as Earth and b) Venusian volcanism must involve either as much water as terrestrial volcanism or have an equally abundant, alternative source of hydrogen. Neither requirements appear plausible.

We note that trace NH_3 can also be produced by some, but not all, volcanic systems on Earth (see (26) and their Table 2.38). For example, (27) reported an emission of volcanic NH_3 from the volcano on Miyake-jima island, in the Izu archipelago, that reached 5 ppb locally. The likely source of NH_3 in such cases is the thermal breakdown of organic matter in the crust that has accumulated in the oceanic sediments. The organic matter reacts with the rising magma beneath the volcano, producing NH_3 gas (27, 28). This mechanism would only apply if there were coal seams on Venus, which itself would be a strong biosignature, albeit one hard to test without drilling.

8. Non-biological pathways of formation of molecular oxygen in the clouds of Venus.

8.1. Production of O_2 by lightning.

The calculation above also suggests that, despite oxygen atoms being abundant in the atmosphere, the production of O_2 is very inefficient and will result in very low abundances of O_2. In addition, any lightning production of O_2 will destroy any > 1 ppt concentrations of NH_3, dissociating the molecule alongside CO_2. The end result will be conversion of the majority of the NH_3 into NO, N_2 and H_2O. Lightning production of O_2 is not compatible with the survival of NH_3.

8.2 Production by thermal breakdown of H_2SO_4.

H_2SO_4 is thermally dissociated into SO_3 and H_2O below ~35 km in Venus atmosphere (29). The equilibrium amount of O_2 formed by the reaction

$$2SO_3 \rightarrow 2SO_2 + O_2,$$

was calculated as follows. The standard free energy of the reaction was calculated from NIST/JANAF tables (17). The abundance of SO_3 is calculated by (30) to be very low in the lower atmosphere, and was taken to be 10^{-7}. The abundance of SO_2 was taken from the same source to be $2x10^{-4}$. From this, the equilibrium partial pressure of O_2 can be calculated for a given pressure. As the pressure for temperatures >730 K (i.e. below the Venusian surface) are speculative, calculations were done at 1 bar – lower pressure will favor O_2 production, and so this favors O_2 production in the lower atmosphere. The results are plotted in Figure S5. This shows that at Venus surface temperature the expected abundance of O_2, if SO_3 is present at the relatively high abundance of 10^{-7}, is ~$5x10^{-12}$, i.e. 6 orders of magnitude less than that reported by Pioneer Venus and required by our model.

Also plotted for comparison is the thermodynamics of the industrial processing pure H_2SO_4, i.e. partial pressure SO_3 = 0.5, and 10% SO_2. This shows that the industrial process is indeed thermodynamically efficient at breaking down SO_3 at temperatures around 700 ^oC.

9. Hydrogen in biological molecules.

We analyzed three databases of biological molecules for the relative abundance of hydrogen atoms. A database of ~200,000 natural products (31) has been exhaustively validated as exclusively being bona fide products of metabolism in a diverse range of organisms. We also used the Roche Biochemical Pathways map of 'core' metabolism (https://www.roche.com/sustainability/philanthropy/science_education/pathways.htm), and the compounds in the KEGG database https://www.genome.jp/kegg/); we note that KEGG contains a number of compounds such as drugs that are not strictly biological molecules. Structures in all three databases were converted into SDF structures with explicit hydrogens using Open Babel (http://openbabel.org/), and the numbers of each atom were counted in each of the databases. These are provided in Table S5, together with the fraction that are hydrogen atoms.

10. Details of the biologically-based NH_3 production model.

Roman numerals refer to elements of the model summarized in Figure 2 of the main text.

(I) NH_3 is produced locally in the clouds from atmospheric N_2 and H_2O (Table 1, main text) by metabolically active microorganisms (black dots) inhabiting cloud droplets (white circle). NH_3 (which is largely confined within the droplet) neutralizes the acid. NH_3 production leads to release of O_2 which diffuses out of the droplet into the atmosphere, therefore contributing to the anomalous detections of O_2 in the clouds.

(II) The production of NH_3 raises the droplet pH to ~1 trapping the SO_2 and H_2O in droplet as ammonium hydrogen sulfite (NH_4HSO_3). The production of sulfite salts in the droplet leads to the formation of a large, semi-solid (and hence non-spherical) Mode 3 particle (white decagon).

(III) The Mode 3 particle settles faster than Mode 2 particles, and falls out of the clouds where ammonium sulfite disproportionates to ammonium sulfate and ammonium sulfide; the latter decomposes to H_2S and NH_3, which in turn undergo photochemical reactions to a variety of products.

(IV) Disproportionation and gas release break up the Mode 3 particles into smaller haze particles. Any microorganisms in the Mode 3 particles would have to form spores (black ovals) to survive in this environment. The data available on the stability of ammonium sulfite or ammonium bisulfite in dry conditions is limited, but suggests the ammonium sulfite disproportionates to ammonium sulfate and ammonium sulfide at ~100 ^{o}C (32); ammonium sulfide is unstable and dissociates to NH_3 and hydrogen sulfide. Survival of spores at 100-120 °C is plausible, based on terrestrial precedent. Some bacteria can even grow at up to 120 °C (in water) (33), and some bacterial spores can survive repeated autoclaving at 135 °C (34); it has even been suggested that some can survive 'ashing' at 420 °C (35).

(V) Some spores can be transported back to the cloud layer by gravity waves, as described by (36). In brief, gravity waves in the atmosphere launched by convective plumes arising in the adjacent (50-55 km and ~18-28 km) convective regions (37–41) can compress atmospheric layers, and allow upwards and downward vertical winds with vertical velocities of ~1 m s^{-1}, as measured directly by the Venera landing probes 9 and 10 at the lower haze layer altitudes (and below) with anemometers (42). Metabolically inactive spores that are brought back to the cloud layers would have to act as cloud condensation nuclei (CCN) and survive in conc. H_2SO_4 long enough to produce a "first batch" of NH_3 to neutralize the acid of the smaller droplet. The mechanism by which this happens is the subject of future work, but possible options are the chemical or photochemical release of NH_3 from stored molecules (which chemically neutralizes the condensing H_2SO_4), the storage of other poly-bases that could neutralize H_2SO_4, possibly forming an ionic liquid phase in which biochemistry could start (e.g. using imidazole derivatives as a base (43)), or that the spores contain highly hygroscopic materials that selectively concentrate water from the environment (perhaps coupled with a selectively sulfuric acid-resistant shell to prevent H_2SO_4 from entering the cell as well (36, 44)). See (36) for more details of this transport mechanism.

(VI) The ammonium sulfate particle falls further below the cloud decks, where ammonium sulfate decomposes to SO_3, NH_3 and H_2O, releasing spores into the sub-cloud haze layer as proposed by (36). Ammonium sulfate decomposes at ~200 ^{o}C to NH_3, water and SO_3 (45).

(VII) Spores released at this stage may be unviable (grey ovals), but any surviving could also be transported back to the clouds.

10.1 Additional commentary on the biologically-based NH_3 neutralization of conc. H_2SO_4 cloud droplets.

For completeness we note that NH_3 may also be consumed in neutralizing the existing sulfuric acid in the cloud droplets to ammonium hydrogen sulfate (NH_4HSO_4). Such scenario could happen if a spore germinates after occupying a pre-formed concentrated sulfuric acid Mode 2 droplet, rather than acting as a CCN and neutralizing acid during the condensation phase (step (V) in the Figure 2 legend). A single cell in an average Mode 2 droplet would take ~6.2 days to convert all the acid in that droplet to ammonium hydrogen sulfate at an NH_3 production rate of ~$4 \cdot 10^{-7}$ grams/gram biomass, assuming 80% sulfuric acid. A typical Mode 2 particle would fall down only ~100 m in ~6.2 days (36). However, we note that the exact kinetics of acid neutralization will depend on the complex kinetics of droplet growth and aggregation.

The scenario above assumes that half of the volume of the typical 2 μm Mode 2 particle is occupied by a cell (1.2 μm in diameter) and the second half is filled with a solvent. The other assumptions and results of calculations are as follows:

- Rate of production (from the literature, as mentioned in the main document) = $2.84 \cdot 10^{-8}$ moles NH_3/gram biomass
- Diameter of mode 2 particle = 2μm
- Volume ≈ $4.19 \cdot 10^{-12}$ cm^3
- Moles of sulfuric acid as pure acid = $5.98 \cdot 10^{-14}$
- Fraction of acid assumed = 80 wt%
- Amount of NH_3 required = 0.8 x $5.98 \cdot 10^{-14} \approx 4.8 \cdot 10^{-14}$ moles
- Mass of biomass in the particle assuming 50% occupancy and a density of 1.5 g/cm^3 = $3.14 \cdot 10^{-12}$ g
- Seconds for that biomass to make that amount of NH_3 = $4.878 \cdot 10^{-14}$ / [$3.14 \cdot 10^{-12}$ x $2.84 \cdot 10^{-8}$] = $5.36 \cdot 10^{5}$ seconds = 6.2 days.

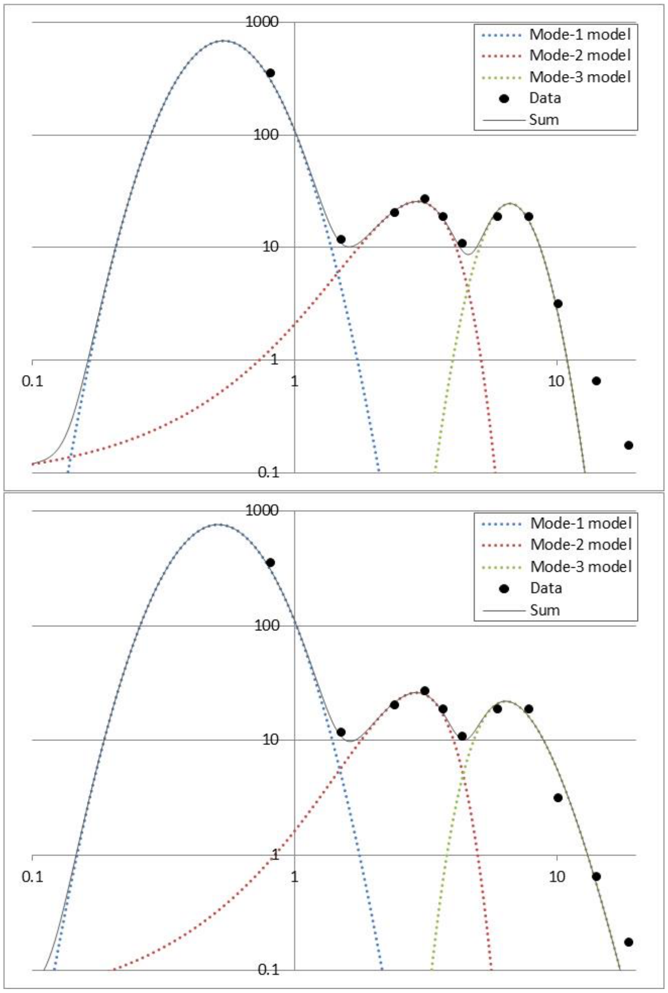

Fig. S1. Observed particle abundances as a function of particle size and trimodal model of those abundances. X axis; particle size in microns. Y axis; abundance in particles/cm^3/µm. Colored dotted lines are models of the three proposed size modes. Solid line is the total distribution. Black dots are data points from the Pioneer Venus sounder probe. Top Panel; match to trimodal distribution where Mode 1 is log-normal, Mode 2 is normal and Mode 3 is log-normal. Bottom Panel: match to trimodal distribution where Mode 1 is log-normal, Mode 2 is normal and Mode 3 is log-log-normal. We adopted a log-log-normal distribution (i.e. a distribution that is normal for log(log(diameter)), a function that is not defined for diameter < 1) for Mode 3 particles, which fits the observed data better (bottom panel).

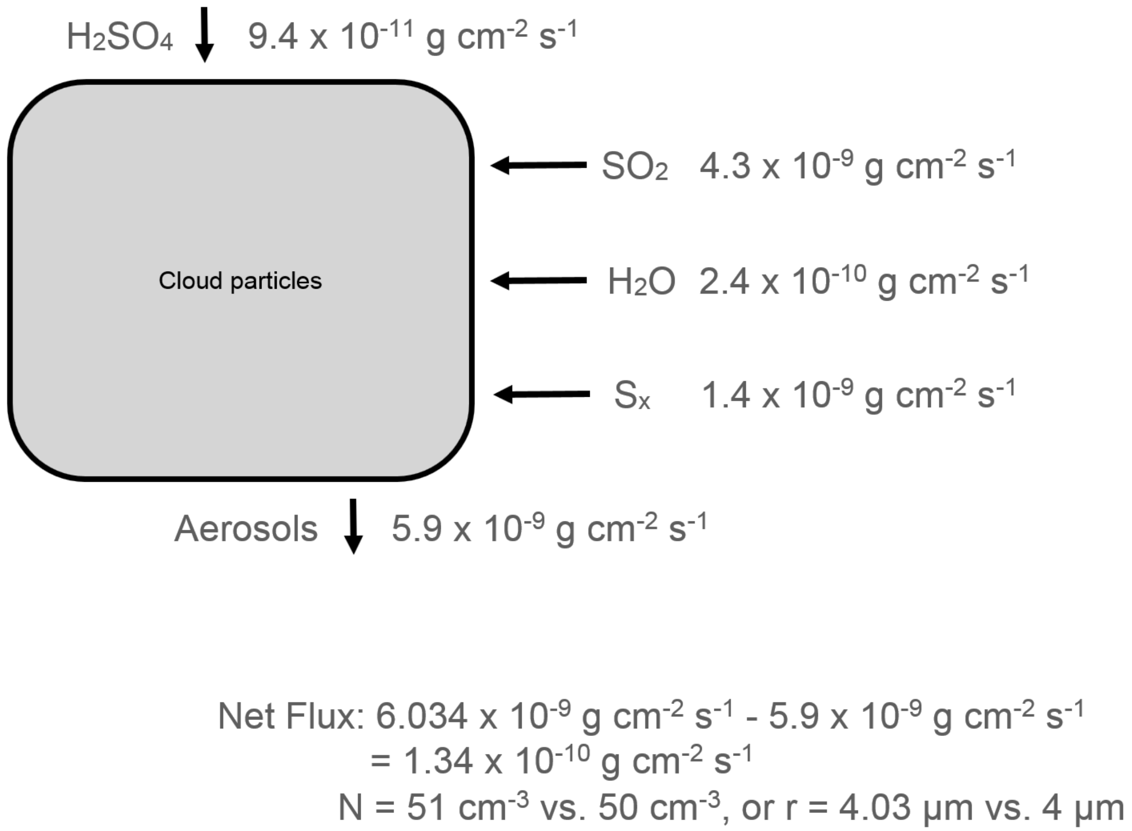

Fig. S2. The overall fluxes of the model.

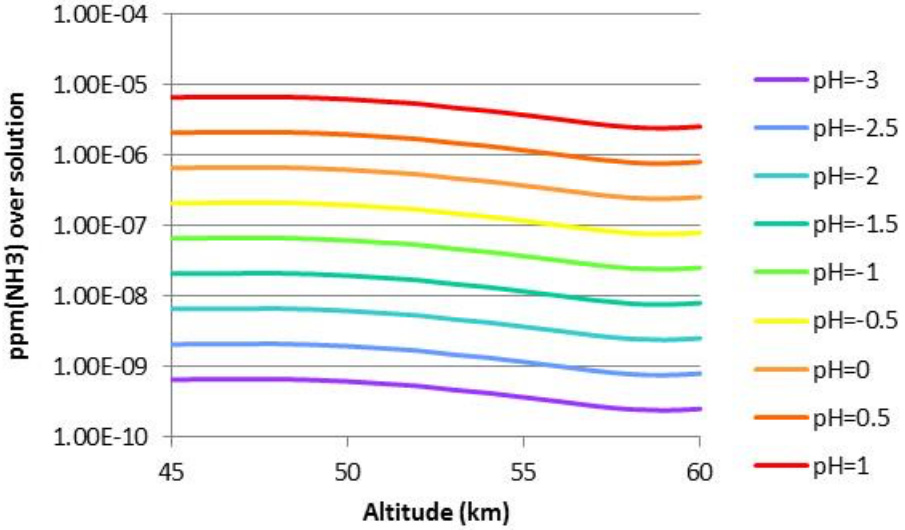

Fig. S3. The vapor pressure of sulfuric acid over ammonium sulfate is in the ppt level at 100 ˚C (46). The detection of vapor phase H_2SO_4 at cloud levels on Venus is likely to be due to the gas phase production of H_2SO_4 from photochemical oxidation of SO_2, as is true on Earth (46).

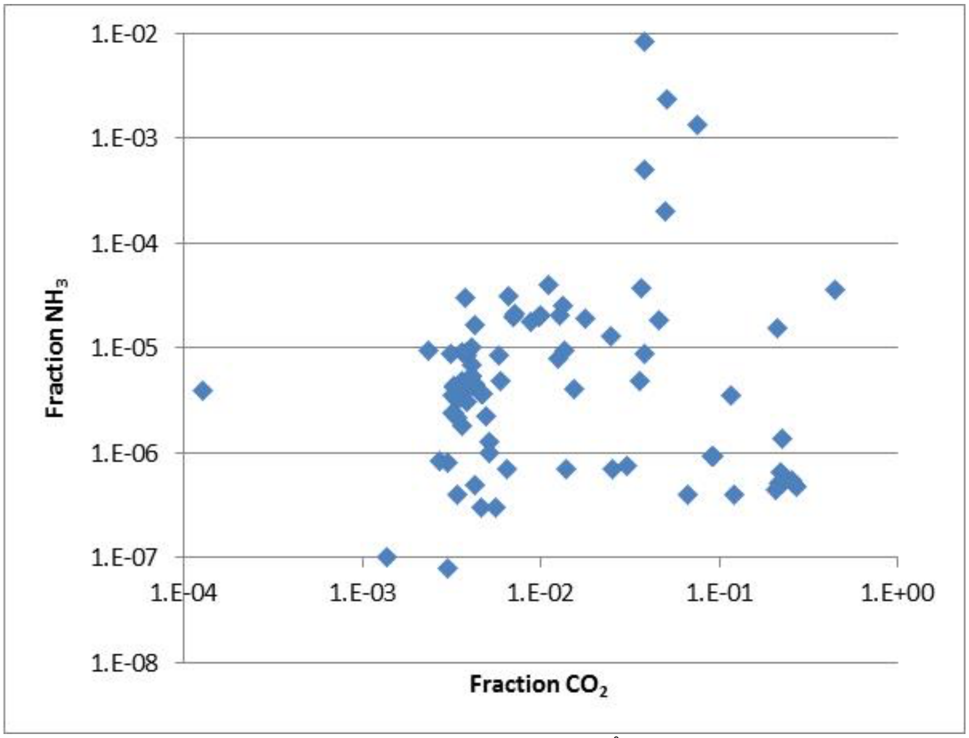

Fig. S4. A tabulation of the fraction of gas over 100 ˚C emitted by terrestrial fumaroles.
Fraction of volcanic gas that is NH_3 (Y axis) vs fraction that is CO_2 (X axis). Data from the following literature sources (47–53).

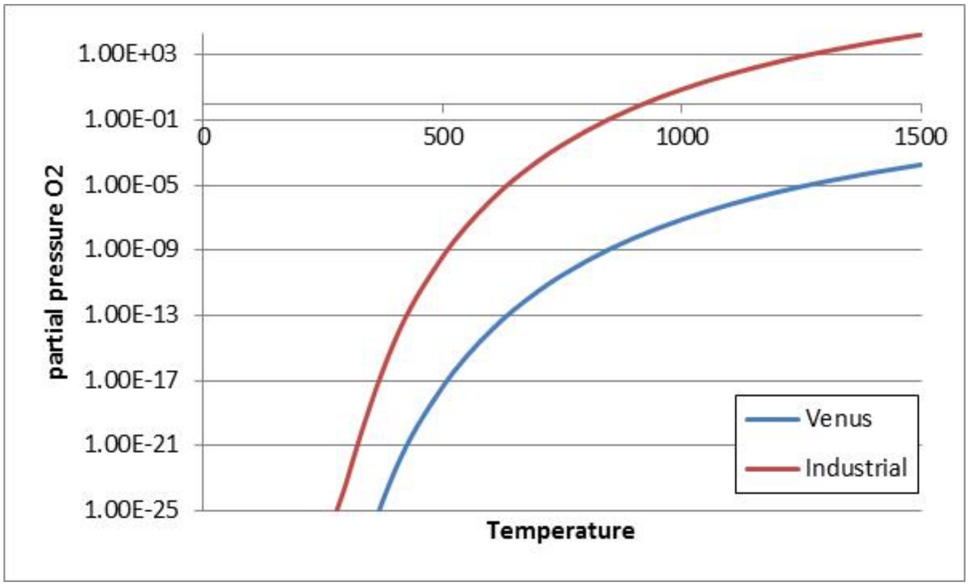

Fig. S5. Prediction of the equilibrium abundance of O_2 formed from the thermal breakdown of sulfur trioxide, at 1 bar pressure, under Venus and industrial gas abundances. See text for details.

Table S1. pKa of SPONCH bases. Only NH_3 has a pKa > the pKa of sulfurous acid of 1.71.

Element	Base equilibrium	pKa
N	$NH_3 \leftrightarrow NH_4^+$	4.75
N	$N_2H_4 \leftrightarrow N_2H_5^+$	5.9
S	$H_2S \leftrightarrow H_3S^+$	<-11
P	$PH_3 \leftrightarrow PH_4^+$	-14

Hydrazine is more reactive, more unstable to photochemical destruction, and requires more energy to synthesize than NH_3, and so we will not consider it further here.

Table S2. The distribution of cloud particles at the altitude of 49 km (i.e. lower clouds). Data was taken from the analysis of the Pioneer Venus sounder measurements reported in (2). The particles have a trimodal distribution.

Mode	Distribution of particle numbers as a function of diameter	Total number per cm^3
Mode 1	Log normal	1200
Mode 2	Normal	50
Mode 3	Log normal	50

Table S3. Assessment of the formation of NH_3 in the Venusian environment by lightning.

Altitude	Temp	Pressure (bar)	Gas phase atoms		Fraction of N as NH_3	Bar of NH_3	moles of NH_3 per cubic meter	Volume of cloud-cloud lightning	Volume cloud-ground lightning	Moles/second
			H atoms	All other atoms						
0	735	92.1	1.266E+24	7.347E+27	5.115E-12	1.649E-11	2.984E-10	0	3.534E+02	1.055E-07
5	697	66.5	9.142E+23	5.305E+27	5.116E-12	1.191E-11	2.273E-10	0	3.534E+02	8.032E-08
10	658	47.39	6.515E+23	3.780E+27	5.115E-12	8.484E-12	1.715E-10	0	3.534E+02	6.063E-08
15	621	33.04	4.684E+23	2.636E+27	5.610E-12	6.488E-12	1.390E-10	0	3.534E+02	4.912E-08
20	579	22.52	3.193E+23	1.796E+27	5.610E-12	4.422E-12	1.016E-10	0	3.534E+02	3.591E-08
25	537	14.93	2.116E+23	1.191E+27	5.609E-12	2.931E-12	7.261E-11	0	3.534E+02	2.566E-08
30	495	9.851	1.338E+23	7.858E+26	4.935E-12	1.701E-12	4.573E-11	0	3.534E+02	1.616E-08
35	453	5.917	8.037E+22	4.720E+26	4.934E-12	1.022E-12	3.001E-11	0	3.534E+02	1.060E-08
40	416	3.501	4.755E+22	2.793E+26	4.932E-12	6.044E-13	1.933E-11	0	3.534E+02	6.831E-09
45	383	1.979	2.687E+22	1.579E+26	4.931E-12	3.416E-13	1.186E-11	7.854E+01	0	9.318E-08
50	348	1.066	1.807E+21	8.501E+25	9.608E-15	3.585E-16	1.370E-14	7.854E+01	0	1.076E-10
55	300	0.5314	2.930E+20	4.237E+25	3.306E-16	6.149E-18	2.727E-16	7.854E+01	0	2.142E-12
60	263	0.2357	6.444E+19	1.879E+25	4.031E-17	3.325E-19	1.682E-17	7.854E+01	0	1.321E-13
65	243	0.0976	2.669E+19	7.787E+24	4.027E-17	1.376E-19	7.534E-18	7.854E+01	0	5.917E-14
70	230	0.0369	1.009E+19	2.942E+24	4.026E-17	5.200E-20	3.008E-18	7.854E+01	0	2.362E-14

Table S4. Calculation of the thermodynamic equilibrium concentration of NH_3 and O_2 if the entire atmosphere was 'shocked' to high temperature and quenched at 1200 K. All properties were evaluated using the Chemkin® Collection and STANJAN (https://navier.engr.colostate.edu/code/code-4/index.html, 2019 David Dandy).

Chemical Equilibrium Results

	Initial State	Equilibrium State
Pressure (atm)	9.8692E+01	9.8692E+01
Temperature (K)	1.2000E+03	1.2000E+03
Volume (cm^3/g)	2.2920E+01	2.2919E+01
Enthalpy (erg/g)	-7.7578E+10	-7.7578E+10
Internal Energy (erg/g)	-7.9870E+10	-7.9870E+10
Entropy (erg/g K)	5.5354E+07	5.5354E+07

	Initial State		Equilibrium State	
	mole fraction	mass fraction	mole fraction	mass fraction
CO2	9.6973E-01	9.8040E-01	9.6973E-01	9.8040E-01
N2	2.9992E-02	1.9301E-02	2.9992E-02	1.9301E-02
H2O	2.9992E-05	1.2412E-05	3.0489E-05	1.2618E-05
SO2	1.4996E-04	2.2069E-04	1.4996E-04	2.2069E-04
CO	9.9972E-05	6.4328E-05	1.0047E-04	6.4649E-05
H2	4.9986E-07	2.3149E-08	2.3128E-09	1.0711E-10
COS	0.0000E+00	0.0000E+00	4.5671E-11	6.3028E-11
SO	0.0000E+00	0.0000E+00	7.3588E-10	8.1251E-10
S2	0.0000E+00	0.0000E+00	3.9969E-15	5.8881E-15
H2S	0.0000E+00	0.0000E+00	3.2978E-14	2.5818E-14
O2	0.0000E+00	0.0000E+00	2.7812E-10	2.0444E-10
CH4	0.0000E+00	0.0000E+00	1.6541E-25	6.0962E-26
NH3	0.0000E+00	0.0000E+00	3.6998E-16	1.4475E-16
NO	0.0000E+00	0.0000E+00	1.5322E-09	1.0561E-09
NO2	0.0000E+00	0.0000E+00	9.0913E-15	9.6082E-15

Table S5. Counts of atoms in all of three databases of biological molecules, the natural products database compiled by (31), the Roche metabolic map database of 'core' metabolism, and KEGG database.

Dataset	Number of molecules	Percentage of atoms that are H	Average molecular weight	C	H	N	O	P	S	Other Elements
Roche map metabolites	699	49.34%	312.97	9056	14337	1205	4028	335	78	16
KEGG	15543	49.01%	392.12	296944	403473	22376	89934	3221	2707	4643
Natural Products Database	204053	49.16%	481.43	5290405	6769512	137557	1545192	1292	11534	13551

Table S6. Boundary conditions for the photochemical model of the Venusian atmosphere.

CO_2	N_2	SO_2	H_2O	CO	OCS	HCl	H_2S	NO	H_2
96%	3%	150 ppm	30 ppm	20 ppm	5 ppm	500 ppb	10 ppb	5.5 ppb	3 ppb

SI References

1. R. Knollenberg, *et al.*, The clouds of Venus: A synthesis report. *J. Geophys. Res. Sp. Phys.* **85**, 8059–8081 (1980).
2. R. G. Knollenberg, D. M. Hunten, The microphysics of the clouds of Venus: Results of the Pioneer Venus particle size spectrometer experiment. *J. Geophys. Res. Sp. Phys.* **85**, 8039–8058 (1980).
3. M. Liler, *Reaction mechanisms in sulphuric acid and other strong acid solutions* (Elsevier, 2012).
4. M. Semmler, B. P. Luo, T. Koop, Densities of liquid H+/NH4+/SO42-/NO3-/H2O solutions at tropospheric temperatures. *Atmos. Environ.* **40**, 467–483 (2006).
5. O. B. Toon, B. Ragent, D. Colburn, J. Blamont, C. Cot, Large, solid particles in the clouds of Venus: Do they exist? *Icarus* **57**, 143–160 (1984).
6. E. P. James, O. B. Toon, G. Schubert, A numerical microphysical model of the condensational Venus cloud. *Icarus* **129**, 147–171 (1997).
7. ChemSpider, ChemSpider: Ammonium Sulfite (2021) (April 20, 2021).
8. P. B. Rimmer, *et al.*, Hydroxide salts in the clouds of Venus: their effect on the sulfur cycle and cloud droplet pH. *Planet. Sci. J.* **2**, 133 (2021).
9. B. Ragent, J. Blamont, Preliminary results of the Pioneer Venus nephelometer experiment. *Science (80-.).* **203**, 790–792 (1979).
10. W. Bains, *et al.*, Evaluating Alternatives to Water as Solvents for Life: The Example of Sulfuric Acid. *Life* **11**, 400 (2021).
11. M. G. Tomasko, L. R. Doose, P. H. Smith, Absorption of sunlight in the atmosphere of Venus. *Science (80-.).* **205**, 80–82 (1979).
12. R. G. Knollenberg, “The response of optical array spectrometers to ice and snow: A study of probe size to crystal mass relationships” (PARTICLE MEASURING SYSTEMS INC BOULDER CO, 1975).
13. L. Esposito, R. Knollenberg, M. Marov, O. Toon, R. Turco, 16. the clouds and hazes of venus. *Venus* **484** (1983).
14. M. Y. Marov, V. Y. Lystev, V. N. Lebedev, The structure and microphysical properties of the clouds of Venus. *USSR Acad. Sci.*, 1–69 (1979).
15. L. V Zasova, V. I. Moroz, V. M. Linkin, Venera-15, 16 and VEGA mission results as sources for improvements of the Venus reference atmosphere. *Adv. Sp. Res.* **17**, 171–180 (1996).
16. J. E. Alleman, Free ammonia-nitrogen calculator & information. *Purdue Univ.* (1998).
17. P. J. Linstrom, W. G. Mallard, The NIST Chemistry WebBook: A Chemical Data Resource on the Internet. *J. Chem. Eng. Data* **46**, 1059–1063 (2001).
18. Y. A. Surkov, B. M. Andrejchikov, O. M. Kalinkina, On the content of ammonia in the Venus atmosphere based on data obtained from Venera 8 automatic station. in *Akademiia Nauk SSSR Doklady*, (1973), pp. 296–298.
19. V. Krasnopolsky, Observation of DCl and upper limit to NH3 on Venus. *Icarus* **219**, 244–249 (2012).
20. I. A. Surkov, B. M. Andreichikov, O. M. Kalinkina, Gas-analysis equipment for the automatic interplanetary stations Venera-4, 5, 6 and 8. *Sp. Sci. Instrum.* **3**, 301–310 (1977).
21. A. T. Young, An improved Venus cloud model. *Icarus* **32**, 1–26 (1977).
22. W. Bains, *et al.*, Phosphine on Venus Cannot be Explained by Conventional Processes. *Astrobiology* **21**, 1277–1304 (2021).
23. J. Huang, S. Seager, J. J. Petkowski, S. Ranjan, Z. Zhan, Assessment of Ammonia as a Biosignature Gas in Exoplanet Atmospheres. *arXiv Prepr. arXiv2107.12424* (2021).
24. K. J. Zahnle, R. Lupu, D. C. Catling, N. Wogan, Creation and evolution of impact-generated reduced atmospheres of early Earth. *Planet. Sci. J.* **1**, 11 (2020).
25. T. M. Gerlach, Present-day CO2 emissions from volcanos. *Eos, Trans. Am. Geophys. Union* **72**, 249–255 (1991).
26. D. M. Möller, *Chemistry of the climate system* (Walter de Gruyter GmbH & Co KG, 2014) https:/doi.org/10.1515/9783110331943.
27. M. Uematsu, *et al.*, Enhancement of primary productivity in the western North Pacific caused by the eruption of the Miyake-jima volcano. *Geophys. Res. Lett.* **31** (2004).
28. H. Sigurdsson, B. Houghton, S. McNutt, H. Rymer, J. Stix, *The Encyclopedia of Volcanoes* (Elsevier, 2015) https:/doi.org/10.1016/c2015-0-00175-7.
29. V. A. Krasnopolsky, Chemical kinetic model for the lower atmosphere of Venus. *Icarus* **191**, 25–37 (2007).
30. M. W. Chase Jr, NIST-JANAF thermochemical tables. *J. Phys. Chem. Ref. Data, Monogr.* **9** (1998).
31. J. J. Petkowski, W. Bains, S. Seager, An apparent binary choice in biochemistry: mutual reactivity implies life chooses thiols or nitrogen-sulfur bonds, but not both. *Astrobiology* **19**, 579–613 (2019).
32. T. Kocsis, *et al.*, Evidence of quasi-intramolecular redox reactions during thermal decomposition of ammonium hydroxodisulfitoferriate (III),(NH 4) 2 [Fe (OH)(SO 3) 2]· H 2 O. *J. Therm. Anal. Calorim.* **132**, 493–502 (2018).

33. K. Takai, *et al.*, Cell proliferation at 122 C and isotopically heavy CH4 production by a hyperthermophilic methanogen under high-pressure cultivation. *Proc. Natl. Acad. Sci.* **105**, 10949–10954 (2008).
34. L. A. O'sullivan, *et al.*, Survival of Desulfotomaculum spores from estuarine sediments after serial autoclaving and high-temperature exposure. *ISME J.* **9**, 922–933 (2015).
35. L. Beladjal, T. Gheysens, J. S. Clegg, M. Amar, J. Mertens, Life from the ashes: survival of dry bacterial spores after very high temperature exposure. *Extremophiles* **22**, 751–759 (2018).
36. S. Seager, *et al.*, The Venusian Lower Atmosphere Haze as a Depot for Desiccated Microbial Life: a Proposed Life Cycle for Persistence of the Venusian Aerial Biosphere. *Astrobiology* **21**, 1206–1223 (2021).
37. A. Seiff, 11. Thermal structure of the atmosphere of Venus. *Venus* **215** (1983).
38. G. Schubert, *et al.*, Structure and circulation of the Venus atmosphere. *J. Geophys. Res. Sp. Phys.* **85**, 8007–8025 (1980).
39. R. D. Baker, G. Schubert, P. W. Jones, Convectively generated internal gravity waves in the lower atmosphere of Venus. Part I: No wind shear. *J. Atmos. Sci.* **57**, 184–199 (2000).
40. R. D. Baker, G. Schubert, P. W. Jones, Convectively generated internal gravity waves in the lower atmosphere of Venus. Part II: Mean wind shear and wave–mean flow interaction. *J. Atmos. Sci.* **57**, 200–215 (2000).
41. M. Lefèvre, S. Lebonnois, A. Spiga, Three-dimensional turbulence-resolving modeling of the Venusian cloud layer and induced gravity waves: Inclusion of complete radiative transfer and wind shear. *J. Geophys. Res. Planets* **123**, 2773–2789 (2018).
42. V. V Kerzhanovich, The atmospheric dynamics of Venus according to Doppler measurements by the Venera entry probes. *Venus*, 766–778 (1983).
43. P. Wasserscheid, M. Sesing, W. Korth, Hydrogensulfate and tetrakis (hydrogensulfato) borate ionic liquids: Synthesis and catalytic application in highly Brønsted-acidic systems for Friedel–Crafts alkylation. *Green Chem.* **4**, 134–138 (2002).
44. D. Schulze-Makuch, D. H. Grinspoon, O. Abbas, L. N. Irwin, M. A. Bullock, A sulfur-based survival strategy for putative phototrophic life in the venusian atmosphere. *Astrobiology* **4**, 11–18 (2004).
45. R. Kiyoura, K. Urano, Mechanism, kinetics, and equilibrium of thermal decomposition of ammonium sulfate. *Ind. Eng. Chem. Process Des. Dev.* **9**, 489–494 (1970).
46. J. J. Marti, *et al.*, H2SO4 vapor pressure of sulfuric acid and ammonium sulfate solutions. *J. Geophys. Res. Atmos.* **102**, 3725–3735 (1997).
47. H. Shinohara, W. F. Giggenbach, K. Kazahaya, J. W. Hedenquist, Geochemistry of volcanic gases and hot springs of Satsuma-Iwojima, Japan: Following Matsuo. *Geochem. J.* **27**, 271–285 (1993).
48. C. J. Janik, M. K. McLaren, Seismicity and fluid geochemistry at Lassen Volcanic National Park, California: Evidence for two circulation cells in the hydrothermal system. *J. Volcanol. Geotherm. Res.* **189**, 257–277 (2010).
49. L. J. Elkins, *et al.*, Tracing nitrogen in volcanic and geothermal volatiles from the Nicaraguan volcanic front. *Geochim. Cosmochim. Acta* **70**, 5215–5235 (2006).
50. I. A. Menyailov, L. P. Nikitina, Chemistry and metal contents of magmatic gases: the new Tolbachik volcanoes case (Kamchatka). *Bull. Volcanol.* **43**, 195–205 (1980).
51. F. Goff, *et al.*, Contrasting hydrothermal activity at Sierra Negra and Alcedo volcanoes, Galapagos Archipelago, Ecuador. *Bull. Volcanol.* **62**, 34–52 (2000).
52. L. E. Clor, T. P. Fischer, D. R. Hilton, Z. D. Sharp, U. Hartono, Volatile and N isotope chemistry of the Molucca Sea collision zone: Tracing source components along the Sangihe Arc, Indonesia. *Geochemistry, Geophys. Geosystems* **6** (2005).
53. Y. A. Taran, V. P. Pilipenko, A. M. Rozhkov, E. A. Vakin, A geochemical model for fumaroles of the Mutnovsky volcano, Kamchatka, USSR. *J. Volcanol. Geotherm. Res.* **49**, 269–283 (1992).
